# Drug resistance mechanisms and treatment strategies mediated by Ubiquitin-Specific Proteases (USPs) in cancers: new directions and therapeutic options

**DOI:** 10.1186/s12943-024-02005-y

**Published:** 2024-05-03

**Authors:** Hongli Gao, Zhuo Xi, Jingwei Dai, Jinqi Xue, Xin Guan, Liang Zhao, Zhiguang Chen, Fei Xing

**Affiliations:** 1 Department of Oncology, Shengjing Hospital of China Medical University, Shenyang, 110004 China; 2 Department of Neurosurgery, Shengjing Hospital of China Medical University, Shenyang, 110004 China; 3 Department of Gastroenterology, Shengjing Hospital of China Medical University, Shenyang, 110004 China; 4 Department of General Surgery, Shengjing Hospital of China Medical University, Shenyang, 110004 China; 5 Department of Emergency Medicine, Shengjing Hospital of China Medical University, Shenyang, 110004 China

**Keywords:** Ubiquitin-specific proteases (USPs), Drug resistance, Immunotherapy, USP inhibitors

## Abstract

Drug resistance represents a significant obstacle in cancer treatment, underscoring the need for the discovery of novel therapeutic targets. Ubiquitin-specific proteases (USPs), a subclass of deubiquitinating enzymes, play a pivotal role in protein deubiquitination. As scientific research advances, USPs have been recognized as key regulators of drug resistance across a spectrum of treatment modalities, including chemotherapy, targeted therapy, immunotherapy, and radiotherapy. This comprehensive review examines the complex relationship between USPs and drug resistance mechanisms, focusing on specific treatment strategies and highlighting the influence of USPs on DNA damage repair, apoptosis, characteristics of cancer stem cells, immune evasion, and other crucial biological functions. Additionally, the review highlights the potential clinical significance of USP inhibitors as a means to counter drug resistance in cancer treatment. By inhibiting particular USP, cancer cells can become more susceptible to a variety of anti-cancer drugs. The integration of USP inhibitors with current anti-cancer therapies offers a promising strategy to circumvent drug resistance. Therefore, this review emphasizes the importance of USPs as viable therapeutic targets and offers insight into fruitful directions for future research and drug development. Targeting USPs presents an effective method to combat drug resistance across various cancer types, leading to enhanced treatment strategies and better patient outcomes.

## Introduction

Cancer poses a significant public health challenge globally, being the first or second leading cause of death in 112 countries [1, 2]. Normal cell growth is controlled by stringent regulatory mechanisms; however, alterations in specific cell components can lead to dysfunction in these mechanisms, resulting in cancer [3]. Cancer treatments are tailored based on the type and stage of cancer, as well as the patient's overall health, including surgical resection, chemotherapy, radiation therapy, targeted therapy, and the burgeoning field of immunotherapy [4]. Despite advancements in molecular and tumor biology that have significantly transformed the cancer treatment landscape and substantially enhanced therapeutic outcomes over the last decades, resistance to treatment continues to be a formidable challenge, particularly in patients with advanced or metastatic disease [5, 6]. Drug resistance, a primary cause of reduced treatment efficacy, is influenced by varied mechanisms [7]. Previous study outlines the key factors of drug resistance, proposing a conceptual framework that encompasses tumor heterogeneity, physical barriers, tumor burden and growth kinetics, undruggable cancer drivers, the immune system and the microenvironment, along with the many consequences of applying therapeutic pressures [8]. Although researches are ongoing to find new drugs and combinations to address drug resistance, the complex molecular mechanisms behind drug resistance remain largely elusive [9]. The identification of new drug resistance biomarkers and a deeper understanding of drug resistance mechanisms are crucial endeavors that will significantly advance personalized precision medicine for cancer treatment [10].

**Fig. 1:**
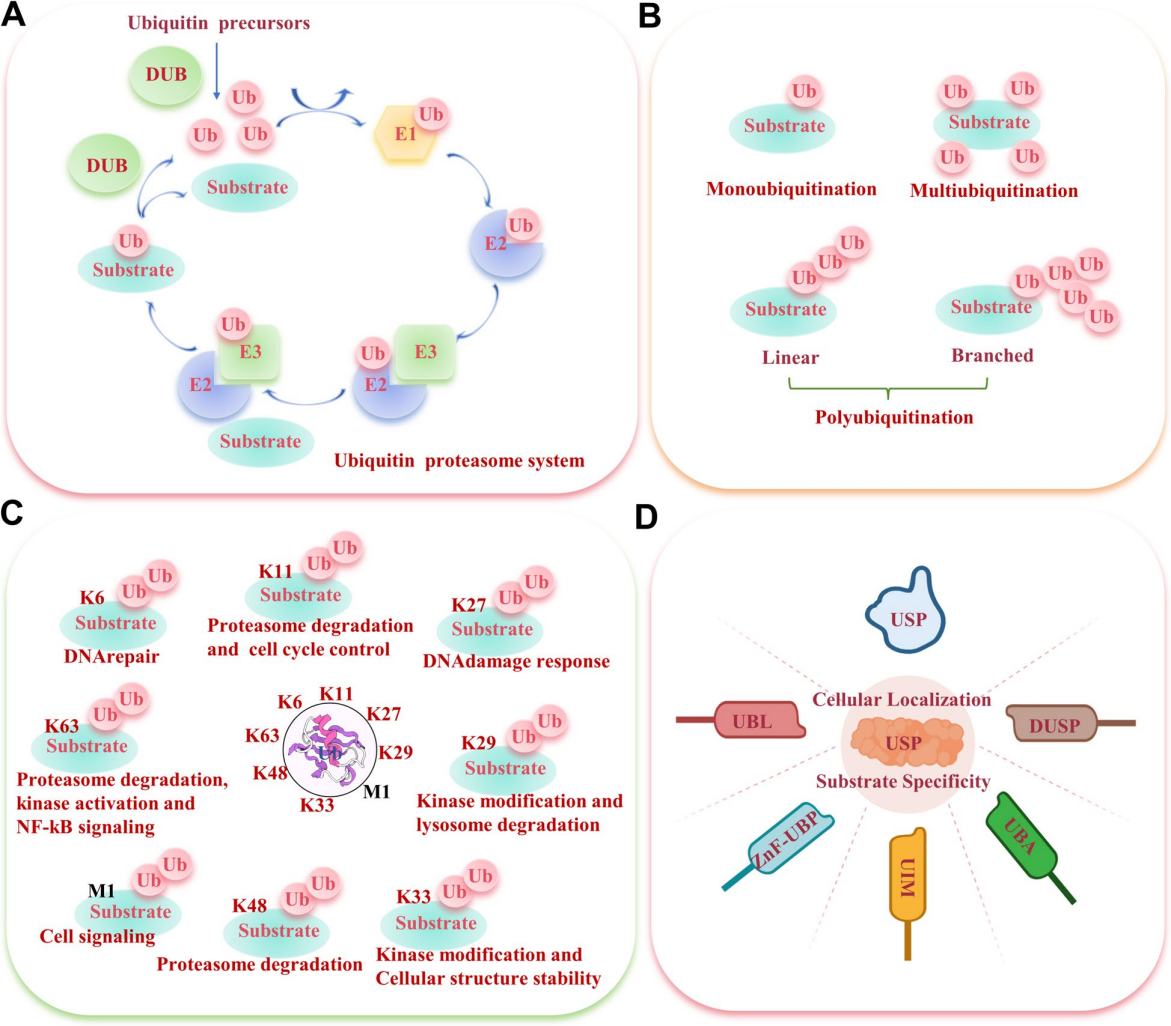
Overview of the ubiquitination and deubiquitination process and their functional implications. **A** The mechanism of the ubiquitin proteasome system. **B** Different types of ubiquitination: monoubiquitination, multiubiquitination, and polyubiquitination. **C** Different substrate fates result from diverse mechanisms of polyubiquitination through the M1 methionine residue or through the seven distinct lysine residues of ubiquitin, K6, K11, K27, K29, K33, K48, and K63. **D** The specific features of USPs involved in biological processes

Post-translational modification (PTM) of proteins is a critical mechanism for modulating protein structure and function in both physiological and pathological conditions, encompassing ubiquitination, phosphorylation, methylation, acetylation, glycosylation, SUMOylation, among others [11]. Ubiquitination, a prevalent form of PTM, involves an ATP-dependent process that attaches ubiquitin to specific proteins. This attachment, involving the 76-amino acid peptide, ubiquitin, initiates protein degradation by the 26S proteasome complex [12]. In recent years, it has become increasingly evident that ubiquitination plays a pivotal role in controlling a broad range of cellular processes beyond protein degradation via the ubiquitin-proteasome system (UPS) (Fig. 1A). Ubiquitin modification acts as a versatile signaling mechanism, regulating protein stability, translocation, signaling activation/inactivation, and even influencing the organization of cellular structures such as organelle membranes and chromatin [13]. The dynamic and precise control of these diverse processes is achieved through the concerted action of a hierarchical enzymatic cascade involving E1 activating enzymes, E2 conjugating enzymes, and E3 ligases. The ubiquitination sequence begins with ubiquitin-activating enzyme E1 binding and activating ubiquitin, followed by the transfer of activated ubiquitin to ubiquitin-conjugating enzyme E2. Then ubiquitin ligase E3 recognizes the substrate and facilitates the transfer of ubiquitin from E2, leading to substrate degradation [14]. E3 ligases, known for their substrate specificity, are crucial in the ubiquitination pathway. The human genome contains approximately 1000 E3 ligases, categorized into RING-between-RING (RBR) family E3s, homology to E6AP C terminus (HECT) domain-containing E3s, and extremely fascinating novel gene (RING) finger domain-containing E3s [15]. These enzymes work together to recognize specific target proteins, transfer ubiquitin molecules, and generate distinct ubiquitin codes, including (i) mono-ubiquitination, where a single ubiquitin molecule is connected; (ii) poly-ubiquitination, forming polyubiquitin chains; (iii) multi-ubiquitination or poly-mono-ubiquitination, with multiple ubiquitin molecules bound [16] (Fig. 1B). In polyubiquitination, ubiquitin is often joined through seven Lysine residues (K6, K11, K27, K29, K33, K48, and K63) and the initial methionine (M1) [17]. These different types of ubiquitin modifications confer specific functional consequences, directing proteins to degradation, influencing protein localization and trafficking, and modulating the activation or inactivation of signaling pathways (Fig. 1C). Moreover, emerging studies have revealed the involvement of ubiquitination in shaping organelle dynamics, regulating membrane fusion events, modulating chromatin structure and DNA repair processes [17]. These findings highlight the multifaceted and intricate roles of ubiquitin in cellular physiology, underscoring its significance as a crucial PTM.

Like other PTMs, ubiquitination is reversible. Deubiquitinating enzymes (DUBs), a type of peptidase, can accurately cleave the C-terminal isopeptide bond of ubiquitin and detach the substrate protein from ubiquitin, thus reversing the ubiquitination process, a phenomenon known as deubiquitination [18]. Ubiquitination and deubiquitination together constitute the complex UPS, which regulates the balance of misfolded proteins in eukaryotic cells. To date, approximately 100 DUB species have been identified in humans, divided into seven subfamilies: ubiquitin-specific proteases (USPs), ubiquitin C-terminal hydrolases (UCHs), ovarian tumor proteases (OTUs), Machado-Joseph disease protein proteases (MJDs), Jab1/Mov34/MPN+ proteases (JAMMs), Zinc Finger ubiquitin-specific proteases (ZUP/ZUFSPs), and motif interacting with ubiquitins (MIUs)-containing novel DUB family members (MINDYs) [19]. The USP family, with over 50 members, is the largest and most diverse, accounting for about 60% of DUBs. USPs are a class of cysteine-dependent proteases, an analogous mechanism of action of the cysteine protease papain, which features three highly conserved subdomains resembling the fingers, thumb, and palm of the right hand [19, 20]. USPs are characterized by the presence of a conserved catalytic domain known as the USP domain, which exhibits protease activity and enables the cleavage of ubiquitin from target proteins. In addition to the USP domain, various USPs possess additional domains or motifs, such as ubiquitin-like (UBL) domain, zinc finger ubiquitin-binding (ZnF-UBP) domain, and domains specific to USP (DUSP), ubiquitin-interacting motifs (UIM) and ubiquitin-associated (UBA), among others [19]. These additional domains influence substrate recognition, protein-protein interactions, and subcellular localization, further augmenting the functional repertoire of USPs. Notably, USPs exhibit diverse substrate specificities, allowing them to target specific ubiquitinated proteins or substrates and regulate distinct signaling pathways and cellular functions [19]. Furthermore, USPs display differential cellular localization, with some USPs predominantly localized in the nucleus, while others are primarily cytoplasmic. This subcellular distribution of USPs contributes to their spatial regulation of protein deubiquitination events [20] (Fig. 1D). USPs control a range of cell processes that are significant in the setting of cancer, including the cell cycle, DNA damage repair mechanisms, chromatin remodeling, and several signaling pathways [17, 18].

In recent years, there has been growing interest in USPs as potential targets for inhibiting tumor formation and cancer progression. So far, over 40 USPs have been connected, either directly or indirectly, to pertinent cancer processes and anti-cancer therapies. The link between USPs and cancer drug resistance is increasingly being substantiated [21]. USPs contribute to drug resistance by catalyzing specific substrate proteins, promoting DNA damage, inducing cancer stem cells (CSCs) characteristics, interfering with cell apoptosis, and regulating transcription factors and key signaling pathways [22]. Gene editing and pharmacological inhibitors targeting USPs could mitigate drug resistance and render cancer cells more vulnerable to anticancer therapies. Current trials investigating the anti-cancer efficacy of USP inhibitors underscore the therapeutic potential of targeting USP-mediated deubiquitination in cancer patients [23, 24]. This review systematically concludes, for the first time, the intricate mechanisms of USP-mediated anticancer resistance across varied treatment modalities, such as chemotherapy, molecular targeted therapy, immunotherapy, and specific radiotherapy. It also explores current potential small molecule USP inhibitors and effective strategies for combining these inhibitors with other anti-cancer means, to modulate drug resistance, aiming to offer innovative approaches and insights for enhancing future cancer treatments.

## Chemotherapy resistance mediated by USPs

### Platinum

After its approval in 1978, cisplatin became a cornerstone in clinical practice as a foundational platinum anticancer drug. A decade later, carboplatin emerged as the second platinum-based drug to be clinically utilized. Then, in 2002, oxaliplatin also successfully entered in Europe and the United States [25]. Despite the advent of precision medicine and immunotherapy, platinum-based treatments, especially cisplatin, remain a mainstay in the treatment of many cancers, serving as the gold standard [26].

#### Cisplatin

##### USPs and DNA damage response (DDR) in cisplatin resistance

The DDR is a highly conserved mechanism that protects cells against DNA damage caused by external and internal factors. It consists of a network of multiple signaling pathways designed to detect and relay damage signals, facilitate damage identification and repair, and ensure the continuation of the normal cell cycle. Unrestricted cyclic DNA replication contributes to unlimited growth and reproduction of cancer cells, and cisplatin exerts its cytotoxic effect by inducing DNA damage and disrupting the protective DDR mechanisms [27]. γH2AX, the phosphorylated form of histone H2AX at Ser139, marks an early cellular response to DNA double-strand breaks (DSBs), initiating and activating the DDR system [28]. In lung cancer patients with cisplatin resistance, the upregulation of USP51 diminishes γH2AX formation and increases checkpoint kinase 1 (CHK1) phosphorylation, thereby ensuring an effective cell cycle [29]. USP22, a crucial regulator that enhances H2AX phosphorylation through its deubiquitinating activity, has been shown to contribute to robust DDR mechanisms in lung adenocarcinoma [30]. Notably, USP22 enhances the repair of DSBs by interacting with the partner and localizer of BRCA2 (PALB2), facilitating the recruitment of the PALB2-BRCA2-Rad51 complex during DDR, ultimately leading to cisplatin resistance [31].

USP7, a typical researched USP member, plays a pivotal role in regulating several key components of DDR pathways, including the MRN-MDC1 complex [32], CHK1 [33], Rad18 [34], RNF168 [35], CDC25A, and p53 [36]. Through its interactions with these proteins, USP7 influences the recruitment of downstream factors involved in DNA damage, modulates the overall functionality of DDR, and confers cellular resistance against genotoxic insults. In the research conducted by Liu et al., USP7 was shown to interact with SAMHD1, a crucial dNTP hydrolase, deubiquitinating it at K421 [37]. This action stabilizes SAMHD1, activating DDR by facilitating further interaction between USP7 and the C-terminal binding protein-interacting protein (CtIP), a key initiator of DSB repair, thus leading to cisplatin resistance [37]. Another significant member, USP1, is regulated at the translational level in cisplatin-resistant non-small cell lung cancer (NSCLC) cell lines [38]. USP1, in a complex with USP1 associated factor 1 (UAF1), removes monoubiquitin from target proteins, FANCD2 and PCNA, which are essential for DDR and chromatin recruitment [39, 40]. Moreover, USP1 can prevent K48-linked polyubiquitination of MAST1, whose overexpression is correlated with increased cisplatin resistance [41, 42]. The loss of USP1 enhances cisplatin-induced DNA damage, evidenced by larger γH2AX foci formation, and diminishes MAST1-mediated activation of phosphorylated MEK/ERK [43].

**Fig. 2:**
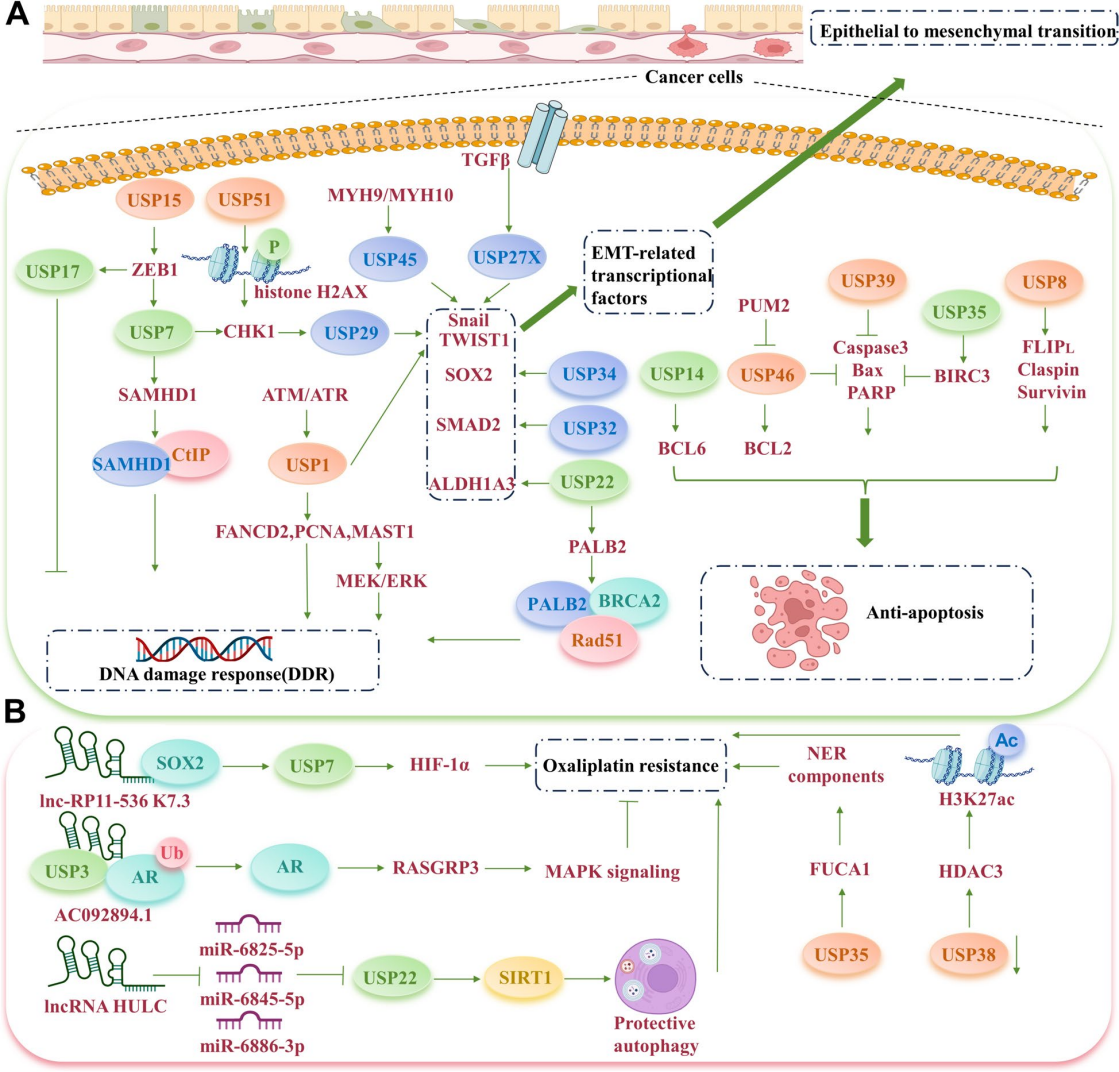
USPs regulate platinum drugs resistance. **A** USPs contribute to cisplatin resistance by regulating DNA damage response, inhibiting apoptosis and enhancing epithelial to mesenchymal transition. **B** Detailed mechanisms of USPs involve in oxaliplatin resistance

Zinc finger E-box binding homeobox 1 (ZEB1) is a key promoter of cisplatin resistance. While ZEB1's role in epithelial-mesenchymal transformation (EMT) and dedifferentiation is well-documented, recent findings also highlight its involvement in enhancing DNA repair and clearance of DSBs [44]. ZEB1 acts as a DNA repair regulator by directly interacting with USP7, thereby augmenting USP7's deubiquitinating activity on CHK1 [45]. Additionally, USP51 can interact with ZEB1, and the reduction of USP51 levels increases ZEB1 ubiquitination, significantly lowering cisplatin resistance in lung cancer cells [46]. On the contrary, overexpression of USP17, a potential downstream target of ZEB1, renders cancer cells more susceptible to cisplatin-induced DNA damage [47] (Fig. 2A).

##### USPs and cell apoptosis in cisplatin resistance

During the DDR, cells may initiate apoptosis to eliminate those with irreparable damage, thereby preventing the proliferation of cells harboring severe errors. Dysregulated apoptosis or evasion of apoptosis constitutes a pivotal mechanism by which cancer cells develop cisplatin resistance [48, 49]. Elevated expression of USP8 in cisplatin-resistant ovarian cancer (OC) cells has been documented. USP8 silencing markedly diminishes the levels of FLIP_L_, Claspin, and survivin, critical regulators of anti-apoptotic pathways [50]. Additionally, USP14's interaction with BCL6, a transcriptional repressor and proto-oncogene, plays a role in anti-apoptotic processes. Inhibition of USP14 effectively mitigates cisplatin resistance in OC cells, enhancing the proteasomal degradation of BCL6 [51]. Furthermore, reduced expression of USP46 contributes to cisplatin resistance by suppressing the apoptotic mediators Caspase3, Bax, and poly‑ADP ribose polymerase (PARP), while concurrently activating BCL2. And this process is potentially under the regulation of PUM2, a Pumilio RNA-binding protein family member [52].

USP39's association with the augmented migratory and invasive capacities of esophageal squamous cell carcinoma (ESCC) cells fosters tumor progression and metastasis. USP39 overexpression impedes PARP and Caspase3 activation, diminishing the apoptotic rate in ESCC cells treated with cisplatin [53]. Beyond ESCC, additional research indicates USP39's regulatory influence on cisplatin-induced apoptosis in colon cancer cells, a process contingent upon the tumor suppressor protein p53. USP39 knockdown escalates p53 levels, enhancing apoptosis and promoting G2/M arrest [54]. Moreover, USP35 stabilizes BIRC3, an apoptosis inhibitor protein (IAP) family member, by averting Lys48-mediated polyubiquitination, impacting PARP and Caspase3 expression in NSCLC cells [55]. Notably, acetylation of USP31 at Lys1264 fosters cervical cancer cell survival and resistance to cisplatin-induced apoptosis. The deacetylase sirtuin 1 (SIRT1) counteracts USP31's oncogenic traits and bolsters cisplatin-induced apoptosis through deacetylation [56] (Fig. 2A).

##### USPs, EMT, and stemness in cisplatin resistance

EMT is a complex biological process transforming epithelial cells into mesenchymal-like cells [57]. CSCs are a subset of tumor cells characterized by pronounced self-renewal capabilities [58]. EMT-induced stemness facilitates the migration of cancer cells from the primary tumor, promotes distant metastasis, and enhances resistance to platinum-based therapies [59]. In triple-negative breast cancer (TNBC) cells, a positive correlation exists between USP22 expression and cisplatin resistance. USP22 overexpression significantly boosts the extracellular acidification rate and spheroid formation while upregulating expression of stemness genes and EMT markers. These unique cellular effects are mediated through USP22's interaction with c-Myc which enhances c-Myc deubiquitination and reduces intracellular glycolysis [60]. In lung adenocarcinoma, USP22 inhibition decreases ALDH1A3 expression, heightens the sensitivity of tumor cells to cisplatin, particularly CD133+ cancer-initiating cells, and attenuates their stem cell-like properties [61].

TWIST and Snail, crucial EMT transcription factors, diminish the chemotherapy sensitivity of cancer cells [62]. USP29-mediated TWIST1 deubiquitination induces cisplatin resistance in TNBC, stabilizing TWIST1 and promoting EMT and CSC activities. CDK1, a USP29 activator, facilitates this process through USP29 phosphorylation, enhancing the TWIST1-driven malignant phenotype [63]. Additionally, USP1, phosphorylated by DDR kinases ATM and ATR, initiates Snail deubiquitination, fostering cisplatin resistance, metastatic potential, and stemness in OC cells [64]. Subsequent studies reveal that, USP45, recruited by MYH9 and MYH10, deubiquitinates Snail in serous ovarian cancer (SOC) [65]. Furthermore, USP27X and Snail expressions are positively linked in breast and pancreatic cancers. During EMT, TGFβ-induced USP27X upregulation stabilizes Snail1 expression in epithelial cells and cancer-associated fibroblasts (CAFs), reducing cisplatin sensitivity [66]. Given TGFβ's role in EMT induction, the regulation of SMAD2, a critical TGFβ pathway component, by USPs is notable [67]. USP32 overexpression in gastric cancer (GC) enhances SMAD2 deubiquitination, correlating with advanced tumor stages, increased cisplatin resistance, and poorer survival [68]. Moreover, in cisplatin-resistant laryngeal squamous cell carcinoma (LSCC) cells, USP34’s interaction with SOX2, a key CSC- and EMT-related transcription factor, decreases SOX2 polyubiquitination and augments LSCC cell sensitivity to cisplatin [69] (Fig. 2A).

#### Oxaliplatin

Oxaliplatin is a fundamental component of FOLFOX, the standardized first-line treatment regimen for gastrointestinal cancers, which also includes 5-fluorouracil (5-Fu) and leucovorin [70]. Recent studies have underscored the pivotal roles of long non-coding RNAs (lncRNAs) in oxaliplatin resistance [71]. The influence of USPs in modulating lncRNAs, and their ensuing effects on oxaliplatin resistance, should not be overlooked.

A recently discovered lncRNA, *lnc-RP11-536 K7.3*, has been found to be associated with oxaliplatin resistance and indicates a poor prognosis in colorectal cancer (CRC) patients. Functionally, *lnc-RP11-536 K7.3* interacts with SOX2, initiating the transcriptional activation of USP7 mRNA. This activation of USP7 facilitates the deubiquitination of hypoxia-inducible factor (HIF-1α), thereby bestowing resistance to oxaliplatin in cancer cells [72]. Conversely, another lncRNA, *AC092894.1*, was found to be significantly downregulated in oxaliplatin-resistant CRC cells. *AC092894.1* serves as a scaffold molecule, enabling the deubiquitination of the androgen receptor (AR) by USP3, fostering the transcription of RASGRP3, and subsequently activating the MAPK signaling pathway, which augments oxaliplatin-induced apoptosis [73]. Moreover, the expression of lncRNA *HULC*, regulated by *miR-6825-5p*, *miR-6845-5p*, and *miR-6886-3p*, elevates the deubiquitination effect of USP22 on SIRT1, making hepatocellular carcinoma (HCC) cells resistant to oxaliplatin and inducing protective autophagy in HCC cells [74].

Given the clinical practice of oxaliplatin combined with 5-Fu, USP-mediated dual resistance to oxaliplatin and 5-Fu has been thoroughly investigated in numerous studies. Upregulation of USP35 promotes CRC cell proliferation and imparts resistance to both oxaliplatin and 5-Fu. Further investigations demonstrated that USP35 directly targets α-L-fucosidase 1 (FUCA1) for deubiquitination, and the USP35-FUCA1 axis elevates nucleotide excision repair (NER) components, culminating in platinum resistance [75]. In contrast, a decrease in USP38 expression was noted in clinical CRC samples, which significantly enhanced the sensitivity of CRC cells to oxaliplatin and 5-Fu. Notably, USP38 plays a crucial role in amplifying oxaliplatin and 5-Fu resistance by removing Lysine 63 ubiquitin chains from histone deacetylase 3 (HDAC3) in CRC cells, accompanied by an increase in H3K27 acetylation [76] (Fig. 2B).

#### Carboplatin

Carboplatin, which is structurally akin to cisplatin, exhibits lower toxicity and fewer side effects than cisplatin; however, resistance remains a challenge [77]. Studies have shown that USP39 protein is overexpressed in carboplatin-resistant OC samples. Mechanistic analyses indicate that USP39 promotes the phosphorylation of AKT, EGFR, and cyclin B1, while it deters the activation of PARP and Caspase-3, thereby enhancing cell proliferation, migration, and invasion, and curbing apoptosis [78]. Additionally, USP48 exhibits high expression in carboplatin-resistant OC cells, too. The reduction of USP48 markedly mitigates chemoresistance to carboplatin and curtails the metastasis of OC cells [79].

### Doxorubicin (adriamycin, dox)

Dox, a member of the anthracycline class, is a prevalent anticancer agent employed in treating various cancers. It exerts its therapeutic effects by intercalating into DNA strands, inducing DNA damage and disrupting DNA replication [80, 81].

#### USPs, cell cycle, and cell apoptosis in Dox resistance

USP7 has been identified as a critical regulator of Dox resistance across several cancer types, including HCC [82], pancreatic ductal adenocarcinoma (PDAC) [83], and neuroblastoma (NB) [84]. In Dox-resistant HCC cells, the disruption of USP7 not only amplifies Dox-induced apoptosis but also impedes cell proliferation via the prolonged activation of the pro-apoptotic protein Bax [82]. In PDAC cells, inhibition of USP7 boosts sensitivity to Dox, correlating with a notable rise in Dox nuclear localization [83]. Additionally, the inhibition of USP7 intensifies the cytotoxic effects of Dox on NB cells, particularly those with an operational USP7-HDM2-p53 axis, increasing their susceptibility to Dox-induced p53-mediated apoptosis [84]. Also in NB, cell viability is influenced by USP14 expression. A synergistic antitumor response is observed when USP14 inhibition is paired with Dox treatment [85].

Bioinformatics analysis has revealed a notable positive correlation between USP37 expression and Dox resistance in BC. The combined approach of USP37 knockdown and Dox treatment significantly increases cleaved Caspase 3 and Bax levels while suppressing BCL2 expression, resulting in cell cycle arrest and enhanced apoptosis [86]. Furthermore, numerous studies have shown that β-transducin repeat-containing protein (β-Trcp)'s regulation of cell cycle depends on its capacity to target Cdc25A [87, 88]. β-Trcp as an E3 ligase engages in specific binding with USP47, and mutations in β-Trcp can impair this interaction. Crucially, disrupting USP47 leads to Cdc25A accumulation, which diminishes cell survival and elevates cellular sensitivity to Dox-induced apoptosis [89]. Additionally, USP8 has been identified as an inhibitor of Dox-induced cell cycle arrest and apoptosis by modulating various receptor tyrosine kinases (RTKs) in HCC, including EGFR, c-Met, p-AKT, p-STAT3, and p-Raf [90].

#### USPs, stemness, and metastasis in Dox resistance

The role of ATP-binding cassette (ABC) transporter-mediated drug efflux is critically examined in TNBC [91]. An increased expression of ABC transporters correlates with resistance to taxanes and anthracyclines, as these drugs, including Dox and paclitaxel, are substrates of p-glycoprotein (Pgp), encoded by the ABCB1 gene [92]. USP7, acting as a specific regulator of ABCB1, engages directly with ABCB1, reducing K48-linked polyubiquitination. Inhibition of USP7 significantly counters resistance to Dox and paclitaxel in TNBC cells, thus diminishing tumorigenesis and distant metastasis in an orthotopic BC mouse model [93]. Additionally, a rise in USP29 expression enhances resistance of NSCLC cells to Dox and paclitaxel by deubiquitinating Snail1 via USP29 [94]. Co-IP assays confirmed that USP45 binds directly to MYC, selectively removing K48-linked ubiquitin chains from MYC, thereby intensifying Dox resistance in cancer cells. The USP45/MYC axis elevates the expression of MYC-targeted downstream factors and CSC-associated proteins, leading to an increase in tumorsphere formation and CD133+ cell populations [95]. Conversely, a notable decrease in USP16 expression was observed in HCC cells. USP16 levels are associated with the carboxyl-terminal truncated form of the Hepatitis B virus X protein (Ct-HBx)-induced upregulation of CSC markers, colony formation, and augmented resistance of HCC cells to Dox [96].

Cell adhesion is integral to the EMT process, and cell adhesion-mediated drug resistance (CAM-DR) is identified as a pivotal mechanism in drug resistance in multiple myeloma (MM) [97]. USP14 is implicated in CAM-DR in MM, where it fosters Dox resistance by inhibiting apoptosis and altering the Wnt signaling pathway [98].

### Paclitaxel

Paclitaxel, a member of the taxane class, influences various cellular oncogenic processes, including mitosis, apoptosis, angiogenesis, inflammatory response, CSC formation, and reactive oxygen species (ROS) production [99, 100]. Notably, as paclitaxel is often used in conjunction with cisplatin or Dox, certain USP-mediated resistance mechanisms previously mentioned may be relevant to paclitaxel resistance as well [63, 93, 94].

#### USPs, cell mitosis, and cell apoptosis in paclitaxel resistance

PLK1 is pivotal in regulating mitosis and orchestrating G2/M cell cycle transition [101, 102]. Recent findings disclose a direct interaction between USP7 and PLK1, with both showing overexpression in paclitaxel-resistant cancer cells. The dual knockdown of USP7 and PLK1 markedly enhances the susceptibility of paclitaxel-resistant cells to apoptosis by influencing chromosome alignment during mitosis [103]. Following this research, targeting USP7 prompts the formation of multiple spindle poles, triggering mitotic catastrophe and apoptosis in lung, prostate, and cervical cancer cells. Synergistic anticancer outcomes are achieved by combining USP7 and PLK1 inhibitors, chiefly through the suppression of MDR/ABCB1 expression [104].

**Fig. 3:**
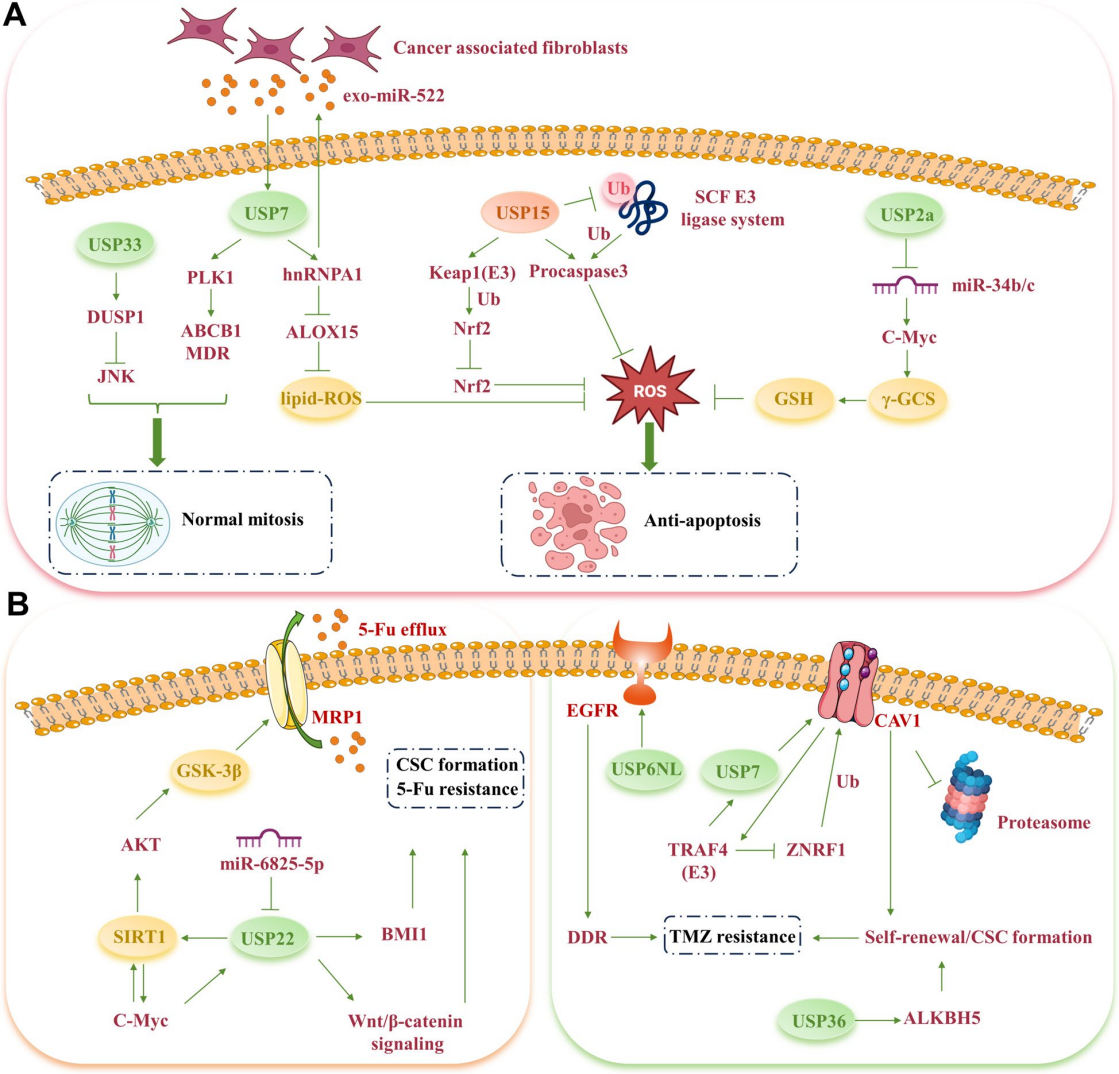
USPs regulate the resistance to paclitaxel, 5-Fu and temozolomide (TMZ). **A** USPs affect paclitaxel resistance by altering cell mitosis, cell apoptosis and reactive oxygen species production. **B** USP22's deubiquitination of SIRT1 and BMI1 supports cancer stem cells formation, aiding in 5-Fu efflux and resistance. **C** USPs participate in regulating TMZ resistance in glioma cells

USP33 overexpression impedes paclitaxel-triggered apoptosis in resistant prostate cancer cells. It interacts with DUSP1, preventing its Lys48-linked polyubiquitination and the subsequent activation of JNK [105]. Intriguingly, Skp1-CUL1-F-box (SCF) E3 ubiquitin ligase system targets procaspase-3, modulating the apoptotic threshold to shield cells from apoptosis [106]. A notable decrease in USP15 expression has been identified in paclitaxel-resistant OC samples. Restoring USP15 expression in paclitaxel-treated cells enhances procaspase-3 deubiquitination, detaches it from the SCF complex, and induces apoptosis, thereby counteracting OC cell resistance to paclitaxel [107] (Fig. 3A).

#### USPs, ROS, and oxidative stress (OS) in paclitaxel resistance

An imbalance between ROS production and antioxidant defense mechanisms triggers OS responses. Paclitaxel promotes ROS generation, which in turn increases OS levels, inducing DNA damage and mutations that contribute to genomic instability and the development of drug-resistant clones [99]. Previously discussed, USP29 upregulation in response to OS stabilizes Snail1 expression, enhancing stemness and resistance to paclitaxel and Dox in NSCLC cells [94]. Crucially, USP2a overexpression in prostate cancer cells reduces ROS production and stabilizes mitochondrial membranes, granting resistance to OS induced by prooxidants like cisplatin, Dox, and paclitaxel [108]. This protective mechanism of USP2a involves regulating c-Myc through *miR-34b/c* to boost intracellular antioxidant glutathione (GSH) levels, thereby mitigating the oxidative cascade initiated by these chemotherapy agents [108].

NF-E2-related factor 2 (Nrf2) is a transcription factor that preserves cellular redox balance by upregulating genes associated with antioxidant response elements (AREs) [109–111]. Zhang et al.'s research demonstrated that USP15 deubiquitinates Keap1, enhancing its E3 ligase activity and prolonging Nrf2 ubiquitination, thus suppressing the Nrf2-dependent antioxidant response. A decrease in USP15 expression elevates Nrf2 levels via a Keap1-dependent pathway, leading to increased paclitaxel resistance [112].

CAFs promote cancer cell growth and drug resistance by releasing various bioactive compounds, including exosomes [113–115]. An intricate study revealed that cisplatin and paclitaxel activate USP7, prompting CAFs to emit exosomal *miR-522*. USP7 then reduces ALOX15 expression by deubiquitinating and stabilizing heterogeneous nuclear ribonucleoprotein A1 (hnRNPA1), diminishing lipid-ROS accumulation and decreasing ferroptosis, ultimately reducing chemotherapy sensitivity in GC cells [113] (Fig. 3A).

### 5‑Fu

5-Fu is a pyrimidine analog classified as an antimetabolite, frequently used alongside other chemotherapy agents. Its primary anticancer action is the noncompetitive inhibition of thymidylate synthase (TS), essential for RNA and DNA synthesis [116].

#### USPs and stemness in 5-Fu resistance

Emerging research indicates that enhanced stemness characteristics mediate 5-Fu resistance in cancer cells. The aforementioned USP16 and USP38 in HCC and CRC also influence 5-Fu resistance by modulating stemness and the expression of related stem cell markers [94, 96]. In recurrent and chemoresistant CRC cells, USP22 expression is elevated, with *miR-305p* identified as an upstream regulator [117]. Inhibiting USP22 expression can adversely affect the Wnt/β-catenin pathway, thus reducing CRC stemness and the cells' resistance to 5-Fu [118]. Furthermore, BMI1, part of the polycomb group (PcG) proteins crucial for stem cell renewal [119], is targeted alongside cisplatin to synergistically suppress growth in head and neck squamous cell carcinoma (HNSCC) cells [120]. It is posited that increased USP22 expression contributes to 5-Fu resistance in HCC cells by elevating BMI1 expression. In a mouse model injected with a 5-Fu-resistant HCC cell line, targeting USP22 led to a significant tumor size reduction post 5-Fu treatment [121] (Fig. 3B).

#### USPs and SIRT1 in 5-Fu resistance

SIRT1, a class III histone deacetylase, serves as an acetylation mediator within the USP22 and SAGA coactivator complex [122, 123]. Studies have demonstrated that USP22 directly interacts with SIRT1, activating the AKT/GSK-3β/multidrug resistance-associated protein 1 (MRP1) pathway, thereby enhancing 5-Fu efflux and reducing 5-Fu-induced apoptosis in HCC cells [124]. Furthermore, a positive feedback loop exists between c-MYC and SIRT1, where USP22 increases SIRT1 stability through MYC mediation, concurrently decreasing p53 levels [125]. Through SIRT1 deubiquitination, USP22 potentially triggers autophagy, diminishing HCC cell sensitivity to chemotherapeutic agents, including 5-Fu [74] (Fig. 3B).

### Temozolomide (TMZ)

TMZ, an orally administered chemotherapy drug, is predominantly used to treat glioblastoma (GBM), an extremely aggressive brain cancer. As an alkylating agent, TMZ induces DNA damage and inhibits cell division [126, 127]. Recent findings indicate that USP4, upregulated in TMZ-resistant GBM cells, inhibits apoptosis in a p53-dependent manner, and this resistance is further amplified by p53-specific inhibitors [103].

Glioma stem cells (GSCs) are a unique population among GBM characterized by their remarkable self-renewal ability and the acquisition of TMZ resistance [128]. USP 6 N-terminal-like protein (USP6NL) is a GTPase-activating protein that plays a regulatory role in EGFR endocytosis [129]. In GBM-resistant cells, the expression levels of USP6NL, as well as CSC markers (CD44 and CD133), transcription factors (Nanog and SOX2), and the efflux transporter ABCG2, were significantly upregulated. Notably, USP6NL was found to interact with EGFR and deubiquitinate it to enhance TMZ-induced autophagy [130]. Additionally, USP36 interacts with and upregulates ALKBH5, an m6A demethylase. Depleting USP36 diminishes GSC self-renewal and increases their sensitivity to TMZ in vitro and in vivo [131]. Significantly, TRAF4, a scaffold protein with E3 ligase activity, binds to Caveolin-1 (CAV1) to inhibit ZNRF1-mediated ubiquitination and facilitate USP7-mediated deubiquitination, thus enhancing CAV1 stability, promoting stemness, and increasing GBM cell resistance to TMZ [132] (Fig. 3C).

## Molecular targeted drug resistance mediated by USPs

### PARP inhibitors

PARP is a critical component of the DDR system, recognizing and binding to DNA single-strand breaks (SSBs), thereby facilitating SSBs repair. Repair of DSBs primarily occurs through two pathways: nonhomologous end-joining (NHEJ) and homologous recombination (HR) [133]. When genes, typically BRCA, responsible for HR at DSBs are mutated, DSB repair is impeded, increasing reliance on PARP-mediated SSBs repair. At the same time, if a PARP inhibitor impedes PARP activity at SSBs, DNA damage cannot be rectified through either SSB or DSB repair mechanisms, leading to cancer cell death. This elucidates why PARP inhibitors are particularly effective in tumor patients with BRCA mutations. Furthermore, PARP inhibitors can be synergistically combined with chemotherapy or radiotherapy to enhance DNA damage in cancer cells [134].

#### USPs and BRCA in olaparib resistance

BRCA1 is pivotal in facilitating HR and is recruited to DSBs through a series of signaling events [135]. Receptor-associated protein 80 (RAP80) plays an essential role in this recruitment, acting through a scaffolding protein to form a complex with BRCA1, thereby promoting the DDR [136]. Recent research indicates that ATM phosphorylation of USP13, following DNA damage, enables USP13 to deubiquitinate RAP80. This action renders OC cells resistant to olaparib by removing K63-linked ubiquitin chains from RAP80 [137]. Additionally, USP15, recruited to DSBs by MDC1, deubiquitinates BARD1 [138, 139], a BRCA1 binding partner, facilitating the interaction between BARD1 and HP1γ at DSBs, thus enhancing olaparib resistance in cancer cells [140].

**Fig. 4:**
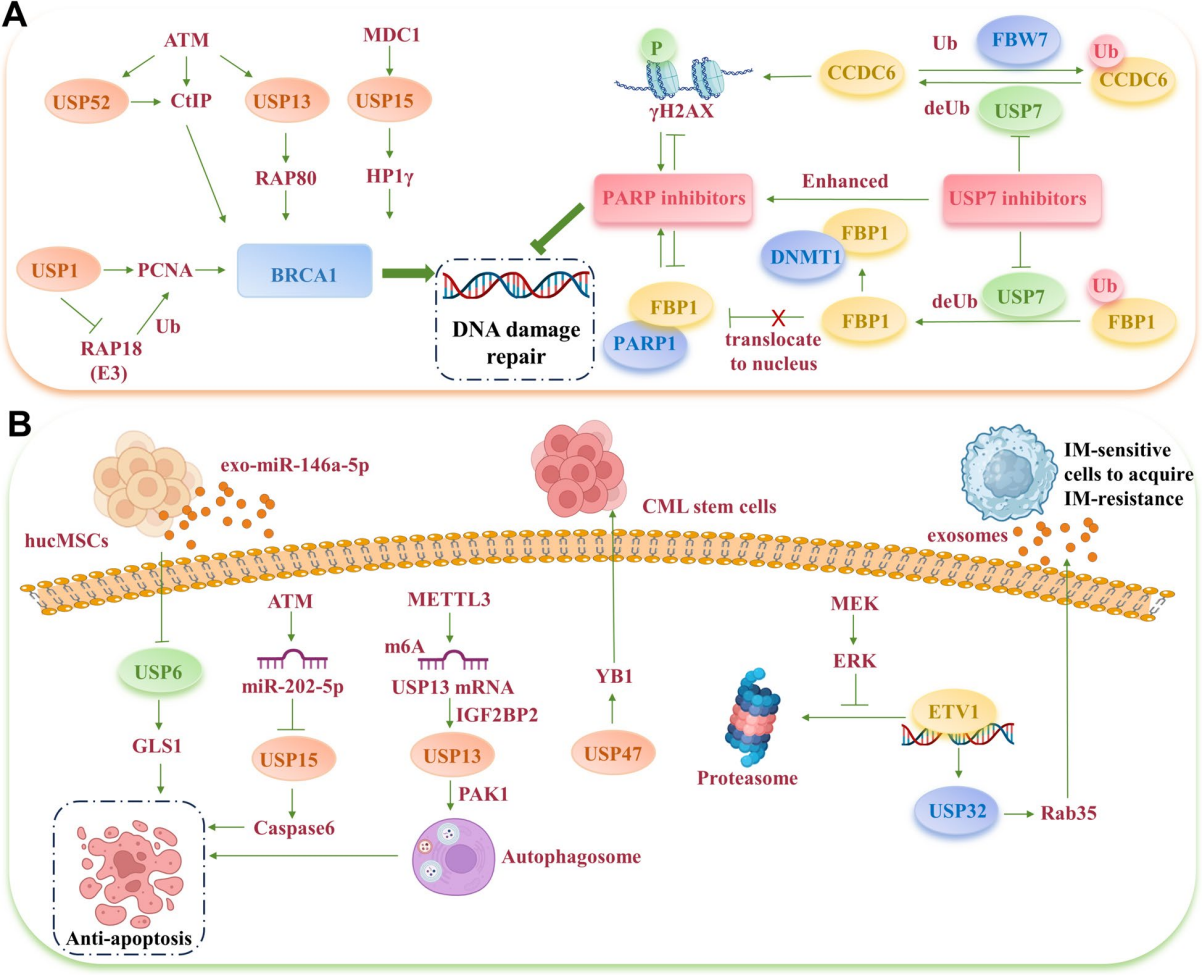
USPs regulate molecular targeted drug resistance. **A** USPs deubiquitinate key nuclear proteins, enhancing DNA damage repair and leading to resistance to PARP inhibitors. Inhibitors targeting USP7 improve the therapeutic efficacy of PARP inhibitors in treating cancer. **B** USPs are involved in imatinib (IM) resistance in chronic myeloid leukemia (CML) and gastrointestinal stromal tumors

CtIP as the key initiator of DDR, also collaborates with BRCA1 to influence olaparib resistance [141]. USP52 can directly deubiquitinate CtIP and facilitate its phosphorylation at Thr-847 [142]. Moreover, in BRCA1-deficient cells, USP1 expression is elevated, leading to its interaction with the essential cell cycle protein PCNA at the replication fork. This interaction prevents PCNA's ubiquitin-mediated degradation by E3 ligase RAD18. In the absence of USP1, persistent loading of the translesion synthesis (TLS) polymerase and the build-up of ubiquitinated PCNA induce replication fork instability, significantly increasing the susceptibility of cancer cells to olaparib [143] (Fig. 4A).

#### USP7 and CCDC6 in Olaparib resistance

USP7 plays a significant role in mediating cancer cell resistance to PARP inhibitors [93, 144–148]. In pancreatic cancer, USP7 deubiquitinates fructose-1,6-bisphosphatase 1 (FBP1) at K206, hindering its nuclear translocation. By preventing FBP1's association with DNA (cytosine-5)-methyltransferase 1 (DNMT1), USP7 inhibits PARP1 entrapment in chromatin, contributing to olaparib resistance [144].

The interaction between USP7 and CCDC6 is crucial in conferring resistance to PARP inhibitors. CCDC6, an ATM substrate, can dephosphorylate γH2AX at S139, maintaining stable DNA damage checkpoints [145]. Studies have identified a positive association between USP7 and CCDC6 expression levels. While the E3 ligase FBXW7 targets CCDC6 for ubiquitination and destabilization, leading to mitotic arrest, USP7's deubiquitination of CCDC6 counters this effect, enhancing its stability and influencing CCDC6 turnover [146]. Inhibiting USP7 promotes CCDC6 degradation, diminishes γH2AX levels, and markedly increases cell sensitivity to PARP inhibitors in various cancer types, including NSCLC [146], prostate cancer [145], lung neuroendocrine cancer [147], bladder cancer [93], and SOC [148] (Fig. 4A).

### Protein kinase inhibitors

#### Tyrosine kinase inhibitors (TKIs)

##### USPs mediate Imatinib (IM) resistance in chronic myeloid leukemia (CML)

IM, a quintessential TKI, is primarily used for treating CML and gastrointestinal stromal tumors (GISTs). CML is a clonal disorder of pluripotent hematopoietic cells, characterized by the presence of a gene, BCR-ABL, that encodes a constitutively active tyrosine kinase fusion protein [149]. IM specifically targets this BCR-ABL protein, significantly inhibiting CML progression [150].

In CML cell lines and peripheral blood mononuclear cells (PBMCs) from CML patients, a reduction in USP15 expression was noted. This decrease in USP15 is due to the upregulation of STAT5A and the direct activation of *miR-202-5p*, which specifically targets and downregulates USP15 mRNA, causing inhibitory deubiquitination of Caspase6 and apoptosis [151]. Furthermore, research has verified an increase in USP6 expression in IM-resistant CML cells [152]. Elevated USP6 levels facilitate the deubiquitination of glutaminase-1 (GLS1), enhancing the conversion of glutamine to glutamate and ammonia, thus impeding IM-induced apoptosis [153]. This pivotal deubiquitination step can be targeted for inhibition by *miR-146a-5p* contained in exosomes from human umbilical cord mesenchymal stem cells (hucMSCs) [152]. Additionally, USP47 is overexpressed in primary CML cells, where it deubiquitinates Y-box binding protein 1 (YB-1). Targeting USP47 presents a promising strategy to counter IM resistance and effectively eradicate leukemia stem/progenitor cells in CML [154] (Fig. 4B).

##### USPs mediate IM resistance in GISTs

GISTs are the predominant malignant mesenchymal tumors of gastrointestinal tract. The c-KIT protein, a common tyrosine kinase in GISTs, is the primary target of IM, particularly the hyperactive mutant form of the c-KIT protein [155]. IM has proven effective in controlling the disease in 70-85% of patients with advanced c-KIT-positive GISTs [156].

The regulation of autophagy-related protein 5 (ATG5) is vital for autophagic activity and IM resistance [157]. USP13 has been shown to deubiquitinate ATG5, thereby enhancing autophagy and increasing IM resistance in GIST cells, a process dependent on serine/threonine-protein kinase PAK1 [158]. The stabilization of USP13 mRNA is facilitated by N6-methyladenosine methyltransferase-like 3 (METTL3) with the aid of the m6A reader IGF2BP2 [158].

Tumor-derived exosomes also play a significant role in mediating IM resistance [159, 160]. Aligning with previous findings, exosomes from hucMSCs and USP6 contribute to IM resistance in CML [152]. Recent studies have indicated that exosomes from IM-resistant GIST cells can confer resistance to IM-sensitive cells, facilitated by Ras-related protein 35 (Rab35). In this context, the transcription factor ETV1 upregulates USP32 expression, which then interacts with Rab35, reducing its K48-ubiquitination and maintaining its stability, thus promoting the resistant mechanism [161] (Fig. 4B).

#### Multiple targeted RTK inhibitors

Sorafenib, a multi-kinase inhibitor, is a recommended treatment for patients with advanced HCC [162]. USP22 plays a role in mediating sorafenib resistance in HCC cells through a complex series of mechanisms. Under normoxic conditions, HIF1α is degraded by the UPS system. However, under hypoxic conditions, HIF1α is stabilized and forms a complex with HIF1β, triggering the transcription of downstream genes [163, 164]. USP22 can enhance hypoxia-induced HCC stemness and glycolysis by deubiquitinating and stabilizing HIF1α. Moreover, USP22 can be transcriptionally upregulated by HIF1α, creating a positive feedback loop that amplifies stemness characteristics and reduces the sensitivity of HCC cells to sorafenib [165]. Notably, a self-activated cascade-responsive nanoplatform, galactose-decorated lipopolyplex (Gal-SLP), has been developed for targeted HCC therapy, facilitating the co-delivery of sorafenib and shUSP22 to achieve a synergistic effect. Sorafenib, encapsulated within Gal-SLPs, initiates a ROS cascade, enabling the rapid release of shUSP22, which inhibits downstream SIRT1/AKT/MRP1 and ABCC1 pathways, increases intracellular sorafenib accumulation, and disrupts glycolysis in HCC cells. This approach demonstrates significant antitumor efficacy and excellent biosafety in a patient-derived xenograft (PDX) model [166, 167].

In addition of USP22, USP29 also deubiquitinates HIF1α, contributing to sorafenib resistance in HCC cells by promoting the transcriptional activation of target genes, especially hexokinase 2 (HK2), a key enzyme in glycolytic pathway [168]. Furthermore, ENKUR, a crucial adaptor protein involved in the localization of a Ca2+-permeable ion channel in sperm [169], is noted for its inhibitory effects on tumor proliferation, metastasis, and sorafenib resistance in HCC. Detailed studies reveal that ENKUR can interact with β-catenin, inhibiting its nuclear translocation and subsequently reducing c-Jun and MYH9 levels. The decreased expression of MYH9 impairs the recruitment of USP7 and the deubiquitination of c-Myc, enhancing the sensitivity of HCC cells to sorafenib treatment [170].

#### Bruton's tyrosine kinase (BTK) inhibitors

Ibrutinib, a prototypical BTK inhibitor, is predominantly employed in treating blood cancers. It targets BTK, an essential component of the B-cell receptor (BCR) signaling pathway, thus impeding B-cell activation, proliferation, and survival [171]. In chronic lymphocytic leukemia (CLL), USP7 is overexpressed and interferes with HR pathways, leading to an accumulation of unrepaired DSBs. Inhibiting USP7 significantly enhances the sensitivity of ibrutinib-resistant CLL cells to clinically achievable doses of chemotherapeutic agents [172]. Additionally, USP14 is implicated in inhibiting tumor-specific apoptosis in ibrutinib-resistant Waldenström macroglobulinemia (WM) cells. The inhibition of USP14 results in the downregulation of BCR-associated elements, disruption of mitochondrial membrane integrity and endoplasmic reticulum stress mechanisms, culminating in increased apoptosis in resistant WM cells [173].

### Receptor inhibitors

#### AR inhibitors

Prostate cancer progression is predominantly driven by AR signaling [174]. Consequently, androgen deprivation therapy (ADT), which diminishes circulating testosterone levels and blocks cellular AR signaling via surgical or chemical castration, remains the cornerstone of treatment for prostate cancer. Based on the response to ADT [175], prostate cancer is classified into hormone-sensitive prostate cancer (HSPC) and castration-resistant prostate cancer (CRPC). Although enzalutamide effectively inhibits AR signaling in CRPC treatment, some CRPC cells evolve to resist enzalutamide by upregulating AR or its splice variant AR-V7 [176].

USP22 is overexpressed in CRPC tumor samples, where it deubiquitinates AR/AR-V7, thereby increasing their accumulation [177]. The lncRNA *PCBP1-AS1* amplifies USP22's deubiquitination effect. Inhibiting *PCBP1-AS1* markedly restores the sensitivity of resistant cells to enzalutamide [178]. Similarly, USP14 deubiquitinates AR/AR-V7 and can outcompete the E3 ligase MDM2, preventing AR's ubiquitination by MDM2 [179]. Kinesin family member 15 (KIF15) facilitates the interaction between USP14 and AR/AR-V7, promoting enzalutamide resistance in prostate cancer cells [180]. Research has identified that nobiletin, a polymethoxylated flavonoid from citrus fruit peels, possesses significant anticancer properties. It induces G0/G1 phase arrest and heightens the sensitivity of AR-V7+ cells to enzalutamide by selectively inhibiting the interactions between AR-V7 and USP14/USP22 [181]. Additionally, glucose-regulated protein 75 (GRP75) hinders the degradation of sinusoidal eye homeobox homolog 1 (SIX1) by facilitating its deubiquitination by USP1. Inhibiting the GRP75-USP1-SIX1 protein complex formation in preclinical models has been shown to delay tumor progression and augment enzalutamide efficacy [182].

#### Estrogen receptor (ER) inhibitors/Endocrine therapy

ER is present in about 70% of breast cancers (BC) and is a pivotal therapeutic target [183]. Patients with ER+ BC benefit from anti-estrogen endocrine therapies, including tamoxifen, an ER antagonist; fulvestrant, an ER modulator; and letrozole, an aromatase inhibitor [184]. Elevated USP22 levels can deubiquitinate ERα, enhancing its transactivation to cis-regulatory elements of ERα target genes, thereby increasing BC cell resistance to tamoxifen [185]. USP15, identified as a novel factor in protecting against ERα degradation, when knocked down, enhances K48-linked ERα ubiquitination, significantly boosting the efficacy of tamoxifen against BC cells [186]. Furthermore, as a crucial component of the PI3K pathway, AKT phosphorylates USP35 at Ser613. The activated USP35 interacts with ERα, boosting its transcriptional activity, which diminishes the effectiveness of tamoxifen and fulvestrant treatments [187].

#### EGFR inhibitors

EGFR-RTK activation plays an important role in the progression of NSCLC. To address this, a series of EGFR-TKI inhibitors, including gefitinib, erlotinib, afatinib, and osimertinib, have been developed specifically for NSCLC patients harboring EGFR mutations [188]. USP8 has emerged as a novel target to counteract gefitinib resistance, with its inhibition leading to the downregulation of multiple RTKs and the induction of cell death in gefitinib-resistant NSCLC cells, while sparing normal cells [189]. In addition to USP8, USP13 inhibits the ubiquitin-mediated degradation of EGFR by the Cbl family of E3 ubiquitin ligases, thereby selectively stabilizing mutant EGFR through a peptidase-independent mechanism [190]. Concurrently, USP22 deubiquitinates EGFR on late endosomes, enhancing its recycling and the sustained activation of various downstream signaling pathways upon EGF stimulation [191]. Moreover, microRNA-124a is identified as a tumor suppressor that targets USP14, reducing stemness and increasing the sensitivity of NSCLC cells to gefitinib [192]. Nonetheless, the precise mechanisms underlying these interactions remain largely unexplored and necessitate further investigation.

#### HER2/ERBB2 inhibitors

HER2-targeted therapies are developed to counteract the overexpression or amplification of HER2 protein in cancers, particularly BC. Trastuzumab, a monoclonal antibody targeting the HER2 receptor, stands out as the most prevalent HER2-targeted treatment. Additional therapies, such as pertuzumab, ado-trastuzumab emtansine (T-DM1), and lapatinib, impede the HER2 pathway through various mechanisms [193]. In the study by Shamshad et al., USP27X was found to be overexpressed in HER2+ resistant BC cells, where it deubiquitinates the CCND1 protein. The ablation of USP27X markedly reduces CCND1 levels and enhances the sensitivity of BC cells to lapatinib [194]. Persistent HER2 protein expression represents a critical resistance mechanism against HER2-targeted therapies. USP2 has been identified as a key regulator of HER2 stability, binding to internalized HER2 to avert its lysosomal degradation. Targeting USP2 reduces HER2 levels by promoting its ubiquitination and degradation [195], offering a potential strategy to overcome resistance in HER2-targeted BC therapies.

### Proteasome inhibitors

Bortezomib (BTZ), a seminal proteasome inhibitor (PI), is extensively employed in the treatment of MM, where it notably impedes NF-κB activation and augments IκBα stability [196]. USP7's role involves deubiquitinating NEK2, thereby stabilizing its expression. Elevated NEK2 levels lead to the binding and phosphorylation of PP1α, initiating the canonical NF-κB pathway and engendering BTZ resistance in MM cells [197]. The ablation of USP7 markedly diminishes colony formation and mitigates BTZ resistance in MM cells by fortifying IκBα expression and obstructing the NF-κB pathway [198–200].

Research consistently shows that autophagy inhibition can significantly slow MM cell growth and induce apoptosis [201]. USP12 emerges as a critical regulator in this context, interacting with and deubiquitinating the autophagy mediator, high mobility group box-1 (HMGB1). The knockdown of USP12 decreases HMGB1 levels, curtails autophagy, and consequently boosts MM cell susceptibility to BTZ [202].

Table 1 encapsulates the described drug resistance mechanisms in cancers, as mediated by the deubiquitination activities of USPs.

**Table 1:** The representative deubiquitination roles of USPs in chemoresistance and molecular targeted drug resistance

Drugs	USPs	Expression levels	Cancer types	Substrate proteins	Biological action mechanisms	Reference
Cisplatin	USP22	Up	Lung Adenocarcinoma	histone H2A	USP22 promotes the phosphorylation of histone H2AX and DDR, decreases the acetylation of Ku70 by stabilizing Sirt1, thus inhibiting Bax-mediated apoptosis.	[30]
	USP22	Up	-	PALB2	USP22 is necessary for BRCA2, PALB2, and Rad51 recruitment to DSBs through stabilizing BRCA2 and PALB2 levels.	[31]
	USP7	Up	-	SAMHD1	USP7 deubiquitinates SAMHD1 at K421, thus stabilizing SAMHD1 to promote DDR by interacting with DSB repair initiator CtIP.	[37]
	USP1	Up	Lung cancer	MAST1	USP1 extends the half-life of MAST1 by preventing its K48-linked polyubiquitination, promotes MAST1-mediated MEK/ERK activation.	[43]
	USP14	Up	Ovarian cancer	BCL6	USP14 interacts with BCL6 to decrease its ubiquitination, enhancing anti-apoptosis.	[51]
	USP35	Up	NSCLC	BIRC3	USP35 interacts with and stabilizes BIRC3 through Lys48-mediated polyubiquitination and alleviates cisplatin-induced cell apoptosis.	[55]
	USP29	Up	TNBC	TWIST1	CDK1-mediated phosphorylation promotes USP29’s deubiquitinase activity toward TWIST1 and TWIST1 driven-EMT and CSC functions.	[63]
	USP1	Up	Ovarian cancer	Snail	USP1 is phosphorylated by ATM and ATR, to deubiquitinates and stabilizes Snail expression.	[64]
	USP45	Up	Serous Ovarian Cancer	Snail	MYH10 combines with MYH9 to recruit USP45 by deubiquitinating Snail.	[65]
	USP27X	Up	Breast cancer and pancreatic cancer	Snail1	USP27X is upregulated by TGFβ and deubiquitinates Snail1 in epithelial cells and CAFs.	[66]
	USP32	Up	Gastric cancer	SMAD2	USP32 enhances SMAD2 deubiquitination, correlating with increased cisplatin resistance, and poorer survival.	[68]
	USP34	Up	Laryngeal squamous cell carcinoma	SOX2	USP34 interacts with SOX2 and reduce its polyubiquitination.	[69]
Oxaliplatin	USP7	-	Colorectal cancer	HIF-1α	Lnc-RP11-536 K7.3 recruited SOX2 to transcriptionally activate USP7 mRNA expression, resulting in deubiquitylation of HIF-1α, facilitating resistance to oxaliplatin.	[72]
	USP3	-	Colorectal cancer	AR	AC092894.1 as a tumor suppressor mediates the deubiquitination of AR through USP3, increasing RASGRP3 transcription and MAPK signaling pathway induced-apoptosis.	[73]
	USP22	-	Hepatocellular carcinoma	SIRT1	lncRNA HULC, regulated by miR-6825-5p, miR-6845-5p, and miR-6886-3p, elevates the deubiquitination effect of USP22 on SIRT1, making HCC cells resistant to oxaliplatin and inducing protective autophagy.	[74]
Oxaliplatin and 5-Fu	USP35	Up	Colorectal cancer	FUCA1	USP35 deubiquitinates FUCA1 to promote NER components expression and drug resistance.	[75]
	USP38	Down	Colorectal cancer	HDAC3	USP38 deubiquitinates HDAC3, increasing H3K27 acetylation and decreasing CSC-related genes expression to demonstrate a tumor suppressor role.	[76]
Doxorubicin and paclitaxel	USP7	Up	TNBC	ABCB1	The N-terminal domain of USP7 directly interacts with ABCB1, reducing K48-linked polyubiquitin chain of ABCB1.	[93]
	USP29	Up	NSCLC	Snail1	USP29 deubiquitinates Snail1 and enhances the cancer stemness.	[94]
Doxorubicin	USP45	Up	Cervical cancer	MYC	USP45 deubiquitinates MYC by removing the K48-linked ubiquitin chain, promoting CSC-related proteins and MYC-targeted proteins expression.	[95]
Paclitaxel	USP7	-	-	PLK1	USP7 deubiquitinates PLK1 protein through its PBD domain and sustains PLK1 protein stability via the C223 site to promote taxane resistance.	[104]
	USP33	Up	Prostate cancer	DUSP1	USP33 interacts with the phosphatase DUSP1, impeding its Lys48 linked polyubiquitination and inhibits JNK activation.	[105]
	USP15	Down	Ovarian cancer	Procaspase3	Decreased USP15 expression lessens procaspase-3 deubiquitination, inhibits its detachment from the SCF complex, suppressing apoptosis, enhancing OC cell resistance to paclitaxel.	[107]
	USP15	Down	Breast cancer	Keap1	USP15 deubiquitinates Keap1and incorporates into the Keap1-Cul3-E3 ligase complex to suppress Nrf2 protein and paclitaxel resistance.	[112]
	USP7	-	Gastric cancer	hnRNPA1	USP7 stabilizes hnRNPA1 to promote miR-522 secretion from CAFs, suppressing ferroptosis and lipid-ROS accumulation, contributing to chemo-resistance.	[113]
5-Fu	USP22	Up	HCC	SIRT1	USP22 directly interacts with SIRT1, leading to the activation of the AKT/GSK-3β/MRP1 pathway, and promoting 5-Fu efflux.	[124]
Temozolomide	USP6NL	Up	Glioblastoma	EGFR	USP6NL deubiquitinates EGFR, promotes the stemness phenotype and DDR, inhibits TMZ-induced autophagy.	[130]
	USP36	Up	Glioblastoma	ALKBH5	USP36 deubiquitinates ALKBH5, a pivotal m6A demethylase, leading to an increase stemness phenotype and self-renewal capacity.	[131]
	USP7	-	Glioblastoma	CAV1	TRAF4-mediated stabilization of CAV1 activates the AKT/ERK1/2 signaling, preventing ZNRF1-mediated ubiquitination and facilitating USP7-mediated deubiquitination, thus maintaining stemness and TMZ resistance.	[132]
Olaparib	USP13	-	Ovarian cancer	RAP80	USP13 is phosphorylated by ATM following DNA damage, then deubiquitinates RAP80 and promotes RAP80-BRCA1 complex recruitment to DSBs and proper DDR.	[137]
	USP15	-	Breast cancer and ovarian cancer	BARD1	USP15 is recruited to DSBs by MDC1 to be phosphorylated at Ser678, then USP15 deubiquitinates BARD1, promotes BARD1-HP1γ interaction, resulting in BRCA1/BARD1 retention and olaparib resistance.	[140]
	USP52	-	Osteosarcoma	CtIP	USP52 is phosphorylated by ATM at Ser-1003 to remove the ubiquitination of CtIP and facilitate the phosphorylation of CtIP at Thr-847, promoting DNA end resection and HR.	[142]
	USP1	-	BRCA1- deficient tumor	PCNA	USP1 inhibits monoubiquitinated PCNA at the replication fork to promote their stabilization in BRCA1-deficient cells.	[143]
	USP7	-	Pancreatic cancer	FBP1	Deubiquitination of FBP1 by USP7 blocks FBP1–DNMT1 interaction, inhibits PARP1 entrapment in chromatin and promotes olaparib resistance.	[144]
	USP7	Up	NSCLC, prostate cancer, lung neuroendocrine cancer, bladder cancer, and serous ovarian carcinoma	CCDC6	USP7 deubiquitinates CCDC6 and promotes HR repair.	[145–148]
Imatinib	USP15	Down	CML	Caspase6	Upregulation of STAT5A and the direct activation of miR-202-5p, which specifically targets and downregulates USP15 mRNA, causes inhibitory deubiquitination of Caspase6 and apoptosis.	[151]
	USP6	Up	CML	GLS1	hucMSC exosomes promotes IM-induced apoptosis by suppressing GLS1 ubiquitination via miR-146a-5p and its target USP6.	[152]
	USP47	Up	CML	YB-1	USP47 facilitates DDR by deubiquitinating YB-1 and promotes TKI resistance.	[154]
	USP13	Up	Gastrointestinal stromal tumors	ATG5	m6A reader IGF2BP2 and METTL3 mediated-USP13 deubiquitinates ATG5, thus enhancing autophagy and promoting IM resistance.	[158]
	USP32	Up	Gastrointestinal stromal tumors	Rab35	ETV1 mediated-USP32 expression deubiquitinates Rab35 at Lys48 and promotes the transmission of IM resistance by enhancing exosome secretion.	[161]
Sorafenib	USP22	Up	HCC	HIF1α	USP22 promotes hypoxia-induced HCC stemness and glycolysis by deubiquitinating HIF1α.	[165]
	USP29	Up	HCC	HIF1α	USP29 deubiquitylates HIF1α to target the encoding HK2 gene and upregulates glycolysis.	[168]
	USP7	-	HCC	c-MYC	ENKUR antagonizes β-catenin/c-Jun/MYH9/USP7 pathway, thus increasing c-Myc ubiquitin degradation and suppressing cell cycle and EMT signals.	[170]
Enzalutamide	USP22	Up	Prostate cancer	AR/AR-V7	LncRNA PCBP1-AS1-mediated AR/AR-V7 deubiquitination by USP22 enhances enzalutamide resistance.	[177]
	USP14	Up	Prostate cancer	AR/AR-V7	KIF15 enhances the AR signaling by binding to AR/AR-V7 and preventing AR/AR-V7 from degradation via increasing the association of USP14 with AR/ARV7.	[180]
	USP1	Up	Prostate cancer	SIX1	GRP75 provides a platform to recruit the USP1 to inhibit K48-linked polyubiquitination of SIX1 to drive castration resistance.	[182]
Tamoxifen	USP22	Up	Breast cancer	ERα	USP22 associates with ERα and enhances the activation of ERα target gene.	[185]
	USP15	Up	Breast cancer	ERα	USP15 enhances the antitumor effects of tamoxifen by promoting K48-linked ERα deubiquitination.	[186]
Tamoxifen and fulvestrant	USP35	Up	Breast cancer	ERα	AKT phosphorylates USP35 at Ser613, leading to its nuclear translocation and subsequent deubiquitination of ERα.	[187]
Gefitinib	USP8	Up	NSCLC	EGFR	USP8 leads to the upregulation of multiple RTKs, including EGFR, and inhibits cell death in gefitinib-resistant NSCLC cells.	[189]
Afatinib	USP13	Up	NSCLC	EGFR	USP13 stabilizes mutant EGFR by counteracting the activity of E3 ubiquitin ligases from the Cbl family.	[190]
Erlotinib	USP22	Up	Lung adenocarcinoma	EGFR	USP22 deubiquitinates EGFR localized on late endosomes, enhances recycling of EGFR after EGF stimulation and activates of multiple EGFR downstream signaling pathways.	[191]
Lapatinib	USP27X	Up	Breast cancer	CCND1	USP27X interacts with and stabilizes the CCND1 protein through deubiquitination to promote cell proliferation.	[194]
Bortezomib	USP7	Up	Multiple myeloma	NEK2	USP7 deubiquitylates NEK2 and activates the canonical NF-κB signaling pathway through the PP1α/AKT axis.	[197]
	USP12	Up	Multiple myeloma	HMGB1	USP12 induces pro-survival autophagy and bortezomib resistance by deubiquitylating HMGB1.	[202]

## Immunotherapy resistance mediated by USPs

Cancer immunotherapy seeks to mobilize the human immune system, utilizing the body's innate ability to eliminate cancer cells [203]. Despite the approval of targeted antibodies against key immune checkpoints, such as programmed death protein-1 (PD-1), programmed death-ligand 1 (PD-L1), and cytotoxic T lymphocyte-associated antigen-4 (CTLA-4) for various cancers, a significant subset of patients encounters resistance and treatment failure [204]. Emerging researches suggest that modulating USP-mediated deubiquitination of proteins in antitumor immune responses may offer a strategy to circumvent immunotherapy resistance [205].

**Fig. 5:**
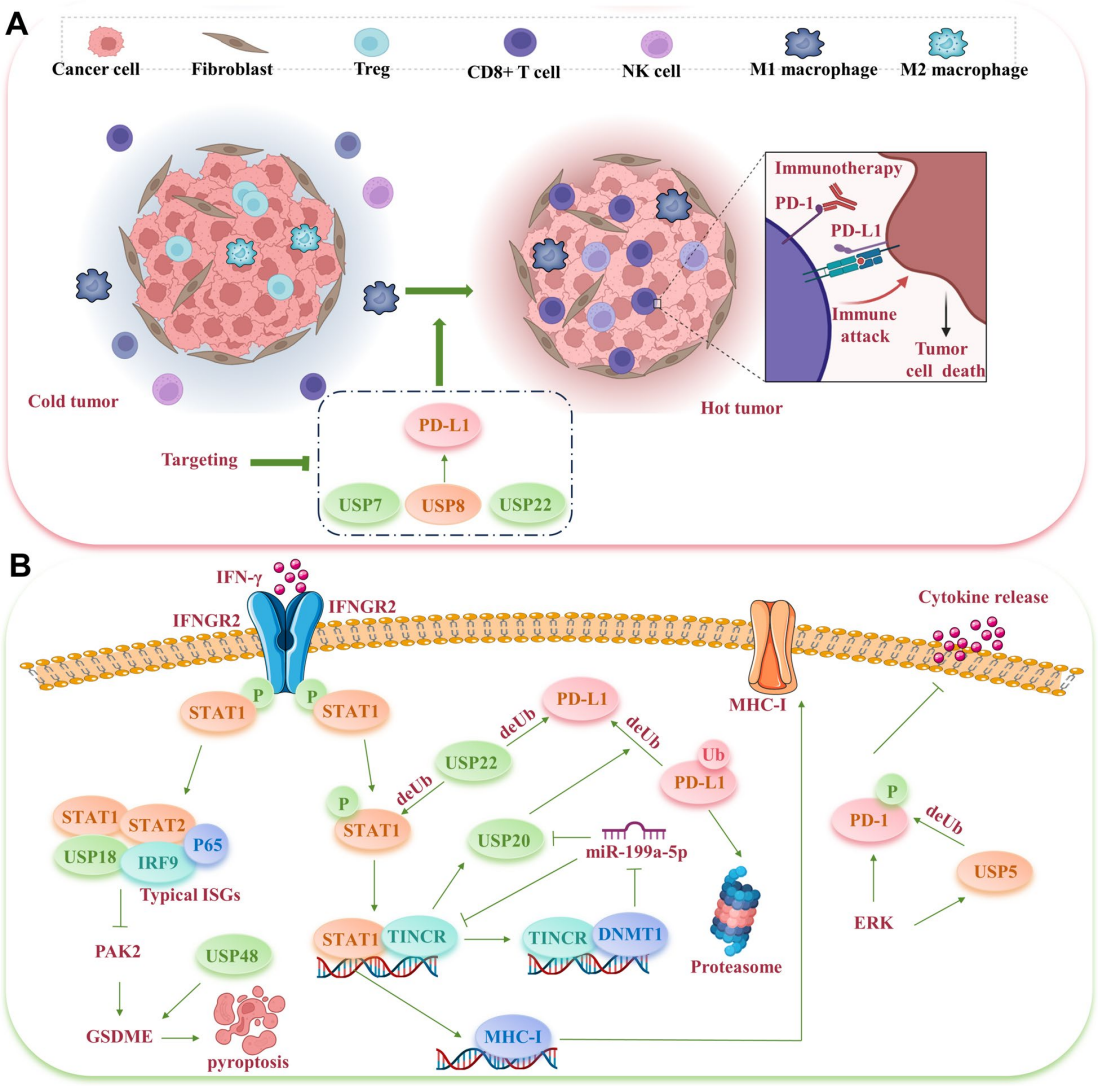
USPs regulate significant cancer immunotherapy resistance. **A** Targeting USP8, USP7, and USP22 affects PD-L1 protein stability, alters immune cells infiltration in tumor microenvironment, and enhances cancer cell sensitivity to immunotherapy. **B** USPs modulate critical IFN signaling pathways to affect cell pyroptosis, MHC-I receptor expression, and cytokine release

Extensive researches indicate the involvement of various USPs in the deubiquitination of PD1/PD-L1 proteins. For instance, USP8, upregulated in pancreatic cancer, can deubiquitinate PD-L1. Targeting USP8 reduces PD-L1’s level, stimulating cytotoxic T-cells, and bolstering the anti-tumor immune response, which enhances the efficacy of PD-L1-targeted immunotherapy [206]. However, a more nuanced study yielded contrary results, indicating that targeting USP8 elevates PD-L1 expression. This increase is primarily due to the intensification of K63 ubiquitination, facilitated by the E3 ligase TRAF6, which counteracts K48 ubiquitination, thereby averting PD-L1 degradation. In this context, USP8 inhibition initiates innate immune responses, boosts IFN type I signaling, and increases MHC-1 production through TRAF6-NF-κB signaling [207]. A similar dichotomy is observed with USP7's influence on PD-L1. In gastric tumors, USP7 suppression diminishes PD-L1 levels, increases the susceptibility of GC cells to T-cell-mediated destruction, and enhances the immune response [208]. Conversely, research by Dai et al. in lung cancer demonstrated that USP7 inhibition might actually intensify PD-L1 expression, associated with greater infiltration of M1 macrophages and IFN-γ+CD8+ T cells, culminating in a robust anti-tumor effect [209]. These disparate findings underscore the complex and context-dependent nature of USP7/USP8's impact on PD-L1. Nevertheless, combining USP8/USP7 inhibitors with PD-1/PD-L1 blockade appears to significantly bolster anti-tumor efficacy (Fig. 5A).

USP22 has been found to interact directly with the C terminus of PD-L1 protein, facilitating its deubiquitination. In a liver cancer mouse model, USP22 knockdown significantly enhanced the efficacy of combined PD-L1 targeted immunotherapy and cisplatin by boosting tumor immunogenicity [210]. In pancreatic cancer, USP22 knockout amplified the response to concurrent anti-PD1 and anti-CTLA4 therapy, notably by diminishing myeloid cell infiltration and encouraging T cell and NK cell presence, thus converting “cold” tumors into “hot” tumors [211] (Fig. 5A). Conversely, melanoma studies indicate that USP22 loss does not enhance immunotherapy effectiveness but rather induces resistance to T cell-mediated cytotoxicity. USP22's ability to deubiquitinate STAT1 and activate the JAK-STAT pathway is crucial; without USP22, STAT1 degradation escalates, inhibiting IFNγ from engaging with its receptors IFNGR1 and IFNGR2, and thus disrupting T cell-mediated cytotoxic signaling [212]. Moreover, IFN-γ activation leads to STAT1 phosphorylation, which triggers its nuclear migration and the subsequent activation of lncRNA TINCR transcription. TINCR then associates with DNMT1, promoting the methylation of *miR-199a-5p* loci and diminishing *miR-199a-5p*'s suppressive effect on USP20, thereby stabilizing USP20 mRNA. Consequently, USP20 deubiquitinates PD-L1, increasing BC cell resistance to PD-L1 inhibitors [213]. Additionally, ERK phosphorylation of PD-1 at Thr234 enables USP5-mediated deubiquitination. Inhibiting USP5 in T cells reduces PD-1 levels, augments effector cytokine production, and decelerates tumor progression in mice, significantly enhancing the response to anti-CTLA-4 or trametinib therapy [214] (Fig. 5B).

Pyroptosis is a distinct form of programmed cell death, differing from apoptosis, characterized as a regulatory necrosis mechanism in inflammatory cells under stress or infection conditions [215]. Researches have shown that pyroptosis plays a crucial role in modulating immunotherapy responses [216, 217]. USP18, by interacting with IFNα receptors and STAT2, diminishes the binding of STAT2-mediated transcription complexes to IFN response elements, thus attenuating type I IFN signaling [218]. Inhibiting USP18 enhances the expression of canonical IFN-stimulated genes (ISGs) and activates a subset of non-traditional ISGs and NF-κB target genes, such as PLK2, leading to the induction of cancer pyroptosis [216]. Gasdermin family member Gasdermin E (GSDME), is activated by Caspase 3, transitioning apoptosis to pyroptosis [219]. USP48 facilitates pyroptosis by binding with GSDME, stripping its K48-linked ubiquitin, and thereby augmenting the functions of T cells and tumor-associated macrophages (TAMs) within the tumor microenvironment (TME), significantly boosting the efficacy of PD-1 inhibitors [218] (Fig. 5B).

Several seminal studies have significantly advanced the understanding of intricate roles of USPs in modulating immunotherapy responses. First, the TET2 DNA dioxygenase is monoubiquitylated at K1299, which augments its enzymatic function and promotes lymphocytes infiltration into tumors [220]. USP15, by removing this monoubiquitin, negatively impacts TET2 activity. Its absence in melanoma cells leads to enhanced IFNγ-induced chemokine production and lymphocytes recruitment, thereby augmenting the immunotherapy responsiveness [221]. Second, the advent of immunomodulatory medicines (IMiDs) like lenalidomide, thalidomide, and pomalidomide has transformed treatment approaches for MM [222]. IMiDs act by binding to cereblon (CRBN), a substrate receptor of the CUL4-RBX1-DDB1-CRBN (CRL4^CRBN^) E3 ligase complex, thereby recruiting neosubstrates as the drug target for ongoing degradation [223]. This study found that USP15 counteracts the CRL4^CRBN^-mediated ubiquitylation of these neosubstrates. Inhibiting USP15 promotes the degradation of these substrates, enhancing the sensitivity of IMiD-resistant MM cells to treatment, and offering a new avenue for CRBN-based PROTACs therapies [224]. Moreover, oncogenic KRAS activation fosters pro-tumorigenic microenvironment [225]. In Kras^G12D^-driven lung cancer, USP12 suppression, triggered by AKT-mTOR signaling, leads to inadequate deubiquitination of PPM1B, resulting in NF-κB signaling hyperactivation and the creation of an immune-suppressive milieu. This environment, characterized by increased macrophage presence, vascularization, and reduced T-cell activity, diminishes the efficacy of anti-PD-1 immunotherapy [226]. Furthermore, the essential amino acid tryptophan's depletion and the rise of kynurenine, catalyzed by IDO1, are crucial for immune evasion [227]. In CRC, USP14 directly deubiquitinates IDO1, shielding it from K48 ubiquitination by TRIM21. USP14 inhibition decreases IDO1 levels, disrupts CD8+ cell activation, alters CD4+ T cell differentiation into Treg cells, boosts the immune response against tumors, and increases the effectiveness of anti-PD-1 treatment [228].

Bioinformatics analysis has demonstrated a significant association between USP35 and an immunosuppressive TME, as indicated by the negative correlation between USP35 levels and CD8+ T cell infiltration in skin cutaneous melanoma [229, 230]. Patients exhibiting high USP35 expression show reduced benefits from immunotherapy compared to those with lower expression levels. A comparable predictive trend is noted for USP51 in GC patients, where increased USP51 expression correlates with decreased immunotherapy efficacy [231]. However, the specific mechanisms through which USP35 and USP51 affect immunotherapy success remain unclear and warrant further experimental investigation.

Table 2 summarizes the resistance mechanisms to cancer immunotherapy mediated by USPs.

**Table 2:** USPs participate in cancer immunotherapy resistance

USPs	Cancer types	Expression levels	Substrates or target proteins	Immunoregulatory mechanisms	Immunotherapy effects	Reference
USP8	Pancreatic cancer	Up	PD-L1	USP8 deubiquitinates PD-L1, and promotes tumor growth by inhibiting cytotoxic T-cells and anti-tumor immune response.	Targeting USP8 can sensitize PD-L1-targeted pancreatic cancer cell to immunotherapy.	[206]
USP8	Melanoma	-	PD-L1	USP8 inhibition increases PD-L1 protein, triggers innate immune response and MHC-I expression through activating the NF-κB signaling.	USP8 inhibition combination with PD-1/PD-L1 blockade activates the infiltrated CD8+ T cells to improve the survival benefit.	[207]
USP7	Gastric cancer	Up	PD-L1	USP7 directly interacts with PD-L1 and stabilize it.	Abrogation of USP7 sensitizes GC cells to T cell mediated killing by downregulating cell surface PD-L1 levels.	[208]
USP7	Lung cancer	Up	PD-L1	USP7 inhibition promotes PD-L1 expression and the infiltration of M1 MΦs and IFN-γ+CD8+T cells by activating the p38 MAPK pathway.	Targeting USP7 inhibits tumor growth and induces anti-tumor immunity.	[209]
USP22	Liver cancer	Up	PD-L1	USP22 directly interacts with the C terminus of PD-L1, leading to its deubiquitination.	Depletion of USP22 improves therapeutic efficacy of CD274-targeted immunotherapy and cisplatin-based chemotherapy.	[210]
USP22	Pancreatic cancer	Up	SAGA/STAGA transcriptional co-activator complex	USP22 inhibits the infiltration of T cells and NK cells by its association with the deubiquitylase module of SAGA/STAGA complex.	Depletion of USP22 enhances the response to the combination therapy of anti-PD1 and anti-CTLA4.	[211]
USP22	Melanoma	-	STAT1	USP22 deubiquitinates STAT1 to regulate IFNγ-JAK1-STAT1 signal axis.	Loss of USP22 contributes to resistance against T cell-mediated cell killing.	[212]
USP20	Breast cancer	-	PD-L1	IFN-γ-phosphorylated STAT1 activates TINCR to recruit DNMT1, then inhibits miR-199a-5p and promotes USP20-mediated PD-L1 deubiquitination.	TINCR impairs the efficacy of immunotherapy via the STAT1-TINCR-USP20-PD-L1 axis.	[213]
USP5	Colorectal cancer	-	PD-1	ERK phosphorylates PD-1 at Thr234 and promotes PD-1 deubiquitination mediated by USP5.	USP5 inhibition in combination with trametinib or anti-CTLA-4 has an additive effect on suppressing tumor growth.	[214]
USP18	Leukemia	-	IFN signaling	USP18 inhibition activates canonical ISGs expression and NF-κB target genes, including PLK2 to trigger cancer pyroptosis.	Targeting USP18 may expand the application of type I IFN treatment to unresponsive cancer subtypes.	[216]
USP48	Pancreatic cancer and HCC	-	GSDME	USP48 promotes pyroptosis by removing K48-linked ubiquitination at K120 and K189.	USP48 enhances the therapeutic efficacy of PD-1 inhibitors by activating the functions of T cells and TAMs within the TME.	[217]
USP15	Melanoma	-	TET2	USP15, acting as a deubiquitinase and inhibitor of TET2 to inhibit TET2 activity and suppress IFNγ-induced chemokine expression and TILs.	Deletion of USP15 leads to increased response to immunotherapy.	[221]
USP15	Multiple myeloma	-	Glutamine synthetase	USP15 antagonizes CRL4^CRBN^-mediated ubiquitylation of glutamine synthetase (GS) and neosubstrates.	Targeting USP15 sensitizes MM cells to immunomodulatory drugs.	[223]
USP12	NSCLC	Down	PPM1B	USP12 downregulation increases macrophage recruitment, hypervascularization, and reduces T cell activity due to insufficient PPM1B deubiquitination.	USP12 inhibition desensitizes lung cancer cells to anti-PD-1 immunotherapy.	[226]
USP14	Colorectal cancer	Up	IDO1	USP14 deubiquitinates IDO1, to activate TRP metabolism, and the conversion of CD4+ T cells into Tregs.	USP14 inhibition contributes to an enhanced antitumor immune response and an increased efficacy of anti-PD-1 therapy.	[228]
USP35	Skin cutaneous melanoma	Up	-	USP35 represents immunosuppressive TME, and is negatively correlated with CD8+ T cells infiltration.	High USP35 expression display reduced benefit from immunotherapy.	[230]
USP51	Gastric cancer	Up	-	USP51 maintains ZEB1 expression to activate M2-like macrophages and fibroblasts.	Elevated USP51 expression exhibits diminished responsiveness to immunotherapy.	[231]

## Radiotherapy resistance mediated by USPs

Radiotherapy, a prevalent cancer treatment modality, employs radiation to induce DNA damage and inhibit cell replication in cancer cells [232]. A key strategy to counteract tumor radioresistance involves disrupting the protective DDR mechanisms. In NSCLC, USP14 modulates DSB repair in response to ionizing radiation (IR) by influencing both NHEJ and HR pathways. Inhibiting USP14 enhances NHEJ efficiency, facilitates the recruitment of essential NHEJ proteins to chromatin, and increases the formation of IR-induced BRCA1 foci [233]. Moreover, radiation triggers the phosphorylation of DGCR8 by the kinase ATM, enhancing DGCR8's deubiquitination by USP51. This enhances the assembly of activated DGCR8 and RNF168 at DSB sites via MDC1, promoting DSB repair and contributing to radioresistance in cancer cells [234].

Histone methylation and acetylation by various enzymes, are crucial in DDR and radioresistance [235]. USP7 facilitates the deubiquitination of histone demethylase PHF8, elevating cyclin A2 levels, which attracts more BLM and KU70 to DSBs, thereby enhancing cellular resistance to radiation [236]. Additionally, USP38 associates with histone deacetylase HDAC1, removes its K63-linked ubiquitin chains, and bolsters the deacetylase activity of HDAC1 on histone H3K56. The absence of USP38 diminishes NHEJ efficiency and heightens cell vulnerability to IR [237].

The CHK family plays a crucial role in regulating cell cycle and mitosis, significantly impacting radiotherapy resistance. In BC cells, USP7 collaborates with *LINC02582* to deubiquitinate and stabilize CHK1, targeting *miR-200c* and enhancing radioresistance [238]. Similarly, USP39 maintains CHK2 stability through deubiquitination. However, its depletion leads to increasing radiation resistance, accompanied with CHK2 dysfunction, impairing the G2/M checkpoint activation after DNA damage and reducing apoptosis [239].

Radiotherapy is a primary treatment modality for GBM, yet resistance to it is common in GBM patients [240]. USP1, highly expressed in GBM and particularly in cells positive for GSC-enrichment markers (CD133 or CD15), modulates the stability of ID1 and CHEK1, which are critical for DDR and stem cell maintenance. Inhibiting USP1 enhances GBM cell radiosensitivity and curtails GSC clonogenic growth and survival [241]. Moreover, USP44's interaction with histone H2B is disrupted by *lincRA1*, which binds to H2B and maintains H2Bub1 levels, impeding USP44's binding, inhibiting autophagy, and fostering radioresistance in GBM [242]. Additionally, the UCH domain of USP3 interacts with the N-terminus of Claspin, stabilizing it against ubiquitination and consequently activating ATR-CHK1 signaling, which contributes to the radioresistance in GBM cells [243].

In addition to the above studies, USPs also play pivotal roles in various pathways, modulating the activity of essential proteins in radioresistance. For instance, USP9X influences apoptosis by targeting MCL-1 [244, 245] or regulates TGFβ signaling via KDM4C [246], while USP7 and USP24 target p53 [247, 248], USP13 targets PTEN [249], USP53 interacts with DNA damage binding protein 2 (DDB2) [250], and USP28 modulates HIF-1α [251]. Due to space constraints, an in-depth discussion of these mechanisms is beyond the scope of this review. For reference, Table 3 succinctly summarizes these mechanisms.

**Table 3:** USPs mediate radiotherapy resistance in cancers

USPs	Cancer types	Expression levels	Substrate proteins	Functional mechanisms to regulate radiotherapy efficacy	Reference
USP14	NSCLC	Up	-	Inhibition USP14 enhances NHEJ efficiency by recruiting of key NHEJ proteins to chromatin, and increasing formation of IR-induced BRCA1 foci, indicating HR deficiency.	[233]
USP51	Breast cancer	-	DGCR8	ATM phosphorylates DGCR8 at Ser 677, facilitating the deubiquitination of DGCR8 by USP51, leading to the recruitment of DGCR8 and RNF168 to MDC1 enabling DSB repair.	[234]
USP7	Breast cancer	-	PHF8	The USP7-mediated PHF8 stabilization confers radiotherapy resistance by recruiting BLM and KU70 to DSB repair.	[236]
USP38	-	-	HDAC1	USP38 deubiquitnates HDAC1 to maintain NHEJ efficiency and increased resistance to IR.	[237]
USP7	Breast cancer	-	CHK1	LINC02582 interacts with USP7 to deubiquitinate CHK1, and target miR-200c, thus promoting radioresistance.	[238]
USP39	Lung cancer	-	CHK2	USP39 deubiquitinates CHK2, repairing DNA damage-induced G2/M checkpoint, increasing apoptosis, and suppressing resistance to radiation treatment.	[239]
USP1	Glioblastoma	Up	-	USP1 promotes radioresistance through maintaining DDR and stem cell maintenance.	[241]
USP44	Glioblastoma	-	H2Bub1	Linc-RA1 inhibits autophagy and promotes radioresistance by preventing H2Bub1/USP44 interaction.	[242]
USP3	Glioblastoma	-	Claspin	Smoothened promotes radiation resistance via activating USP3-mediated claspin deubiquitination and ATR-CHK1 signaling.	[243]
USP9X	Lung cancer	-	KDM4C	USP9X-mediated KDM4C deubiquitination promotes radioresistance by epigenetically inducing TGF-β2 transcription and activating Smad/ATM/Chk2 signaling.	[246]
USP24	-	-	P53	USP24 is a p53 deubiquitinase, and promotes PUMA activation and inhibits cell resistant to apoptosis after UV damage.	[248]
USP13	Oral squamous cell carcinoma	-	PTEN	Bergenin upregulates the PTEN protein by enhancing the interaction between PTEN and USP13, thus inhibiting glycolysis and overcoming radioresistance.	[249]
USP28	Esophageal cancer	Up	c‐Myc	Knockdown of USP28 enhances the radiosensitivity of via destabilizing c‐Myc and enhancing the accumulation of HIF‐1α.	[251]

## Overcome anti-cancer drug resistance by USP inhibitors

Recent advances in USP inhibitors as therapeutic agents have demonstrated significant anti-cancer potential, with extensive reviews covering their development and clinical applications [14, 252, 253]. This section highlights USP inhibitors crucial for overcoming drug resistance in cancer treatment (Table 4).

**Table 4:** USP inhibitors involve in overcoming drug resistance of cancers

Target	Inhibitor	Chemical structure	Enzyme activity/IC50	Cancer type	Biological mechanism in drug resistance	Reference
USP7	P22077		USP7/USP47 (8µM)	Neuro-blastoma	P22077 enhances the cytotoxic effects of Dox and etoposide in NB cells with an intact USP7-HDM2-p53 axis.	[84]
				HCC	P22077 induces cell death, inhibits cell proliferation and migration and decreases cell sensitivity to chemotherapy.	[82]
				Pancreatic cancer	P22077 reduces protein synthesis, and alters the extracellular space matrix to overcome Dox resistance.	[83]
				Acute myeloid leukemia	P22077 reduces cell proliferation, blocks DNA replication progression and increases the killing effect of cytarabine.	[254]
				Lung cancer	Combination treatment with the mitotic kinase PLK1 inhibitor volasertib and the P22077 shows a strong synergism through down-regulation of MDR1/ABCB1 in paclitaxel-resistant lung cancer.	[104]
				Chronic lymphocytic leukemia	P22077 eliminates leukemia stem/progenitor cells and overcomes imatinib resistance through destabilizing YB-1 and inhibiting DDR.	[154]
	P5091		USP7(4.2µM)	Multiple myeloma	P5091 induces apoptosis in MM cells and overcomes BTZ resistance. Combining P5091 with lenalidomide, HDAC inhibitor SAHA, or dexamethasone triggers synergistic anti-MM activity.	[177]
				Multiple myeloma	P5091 and NEK2 inhibitor together overcome BTZ resistance through regulating NF-κB signaling pathway and the PP1α/AKT axis.	[175]
				Multiple myeloma	RRx-001 plus P5091 triggered synergistic anti-MM activity and overcome BTZ resistance.	[255]
				Lung neuro-endocrine tumor	P5091 sensitizes lung neuroendocrine tumor cells to PARP inhibitor by lowering CCDC6 and HR repair.	[147]
				Prostate cancer	P5091 accelerates the degradation of AR and CCDC6, sensitizing cancer cells to PARP-inhibitors.	[145]
				Lewis lung carcinoma	P5091 upregulates PD-L1, deregulates PD-1 and reprogramming TAMs in TME, enhancing the anti-tumor immune response.	[209]
	GNE6776		USP7(1.34µM)	TNBC	GNE-6776 increased apoptosis in chemoresistant TNBC cells through inhibiting the interaction between USP7 and ABCB1.	[93]
	HBX19818		USP7(4.2µM)	Chronic lymphocytic leukemia	P22077 sensitizes p53-defective, chemoresistant CLL cells to chemotherapeutic agents through the accumulation of DNA damage.	[172]
	Compound41		USP7(0.44nM)	NSCLC	Compound 41 resensitizes MYCN-overexpressing chemoresistant NSCLC cells to cisplatin and etoposide treatment by decreasing N-MYC protein and increasing apoptosis.	[256]
USP1	GW7647		USP1/UAF1 (5µM)	NSCLC	GW7647 in combination with cisplatin together enhances monoubiquitylation of PCNA and FANCD2 to promote therapeutic efficacy.	[40]
	Pimozide		USP1/UAF1 (1.96µM)	Lung cancer	The combination of pimozide and MAST1 inhibitor lestaurtinib sensitizes cells to cisplatin by reducing MAST1 expression and subsequent phosphorylation of MEK1 and ERK.	[43]
				B-cell lymphoma	Pimozide shows a synergetic effect with etoposide in rituximab/chemotherapy resistant cells through destabilization of MAX.	[257]
	SJB3-019A (SJB)		USP1/UAF1 (781nM)	Multiple myeloma	Combining SJB with the ACY-1215, BTZ, lenalidomide, or pomalidomide shows synergistic cytotoxicity through activating apoptosis, inhibiting DNA repair and HR, as well as the downregulating stem cell renewal.	[258]
	ML323		USP1/UAF1 (76nM)	NSCLC	ML323 enhances the cytotoxicity of cisplatin in resistant NSCLC by inhibiting the deubiquitylation of PCNA and FANCD2.	[38]
				Breast cancer and ovarian cancer	ML323 kills BRCA1 deficient cells that have acquired resistance to PARP inhibitors due to replication fork stabilization.	[143]
USP13	Spautin-1		USP10/USP13, (0.6-0.7µM)	Ovarian cancer	Spautin-1 disrupts the formation of RAP80-BRCA1 complex foci and impairs DDR, thus rendering cancer cells sensitive to olaparib.	[137]
				Gastro-intestinal stromal tumor	Spautin-1 induces the decay of ATG5 and co-administration of spautin-1 with 3-methyladenine enhances the therapeutic efficacy of imatinib.	[158]
USP14	b-AP15		USP14/UCHL5 (2.1µM)	Waldenström macro-globulinemia	b-AP15 can induce apoptosis in cells overexpressing Bcl-2 and lacking functional p53 to overcome BTZ resistance.	[259]
	VLX1570		USP14/UCHL5 (10µM)	Waldenström macro-globulinemia	VX1570 promotes rapid and tumor-specific apoptosis in WM cells resistant to BTZ or ibrutinib.	[173]
	IU-1		USP14(4.7µM)	Colorectal cancer	IU1 combined with anti-PD-1 enhances the anti-tumor response through regulating the infiltration ratio of CD8+ T cells and FOXP3+ Treg cells, also inhibiting IDO1-mediated immune suppression.	[228]
USP8	DUBs-IN-2		USP8(0.93µM)	-	DUBs-IN-2 leads to the upregulation of PD-L1, triggering immune responses and antigen presentation through TRAF6-NF-κB signaling pathway to enhance the efficacy of anti-PD-1/PD-L1 immunotherapy.	[207]
				Pancreatic cancer	The combination therapy of DUBs-IN-2 and anti-PD-L1 suppress tumor growth, which is mediated by the activating anti-tumor immunity relies on the PD-L1 pathway and CD8+ T cells.	[206]
	9-ethyloxyimino-9H-indeno[1,2-b] pyrazine-2,3-dicarbonitrile		USP8(<1µM)	NSCLC	This inhibitor suppresses the expression of multiple RTKs in gefitinib-resistant NSCLC cells by enhancing the colocalization between Ub and target RTKs.	[196]
				HCC	This inhibitor enhances the efficacy of DOX or sorafenib by reducing the expression levels of RTKs.	[90]

### USP7 inhibitors

Among USP inhibitors, USP7 inhibitors are the most varied and thoroughly researched. The thiophenyl compound P22077, a notable USP7 inhibitor, induces apoptosis by targeting USP7 and enhancing intracellular ROS production [260]. It stabilizes p53 and degrades HDM2, augmenting the cytotoxic effects of Dox and etoposide on NB cells [84]. In HCC and PDAC, P22077 lessens the cells' sensitivity to Dox [82, 83]. Additionally, P22077 disrupts the USP7-CHK1 interaction, aiding in overcoming cytarabine resistance in AML [254]. The combination of P22077 with the PLK1 inhibitor volasertib shows synergistic efficacy in paclitaxel-resistant lung cancer [104]. Interestingly, P22077 not only targets USP7 but also addresses IM resistance in CML by inhibiting USP47, enhancing the effectiveness against TKI-resistant CML cells and reducing Lin^−^Sca1^+^c-Kit^+^ CML stem/progenitor cell numbers in CML models [154].

Through high-throughput screening, scientists identified another novel USP7 inhibitor, the thiophenyl compound P5091, which induces apoptosis in BTZ-resistant MM cells. When combined with lenalidomide, dexamethasone, or SAHA (an HDAC inhibitor), P5091 demonstrates synergistic therapeutic effects [177]. In MM cells, the concurrent use of the NEK2 inhibitor INH1 and P5091 markedly impedes cell growth and overcomes NEK2-related and inherent BTZ resistance by modulating the NF-κB and PP1α/AKT pathways [175]. Moreover, the hypoxia-selective epigenetic agent RRx-001 triggers MM cell apoptosis through Caspase activation, increased ROS release, and reduced global methylation, exhibiting synergistic anti-MM effects with P5091 in overcoming BTZ resistance [255]. P5091 also enhances the sensitivity of lung neuroendocrine tumor cells to PARP inhibitors by diminishing CCDC6 levels and hampering HR repair, showing combined efficacy against lung neuroendocrine and CRPC [145, 147]. As for immunotherapy, P5091 escalates PD-L1 expression, while it blocks PD-1 and reprograms TAMs in TME, facilitating an effective antitumor response in Lewis lung carcinoma [209].

GNE-6776, another prominent USP7 inhibitor, exhibits significant inhibitory activity against the USP7 catalytic domain [261], markedly increasing apoptosis in chemoresistant TNBC cells [93]. HBX19818, which covalently binds to USP7's active site, enhances the sensitivity of chemoresistant and p53-deficient CLL cells to chemotherapy [172]. Notably, RAPT Therapeutics, Inc. has developed a unique USP7 inhibitor, compound 41 [262], which re-sensitizes MYCN-amplified chemoresistant tumors to cisplatin and etoposide by reducing N-MYC levels and increasing cleaved Caspase 3 [256].

### USP1 inhibitors

Given the functional role of USP1 as part of the USP1/UAF1 complex, extensive researches have been conducted to develop inhibitors targeting this complex. In 2011, the first USP1/UAF1 inhibitor was identified through a high-throughput screening using Ub-Rho110 [40]. After that, pimozide and GW7647, identified as the most effective compounds, demonstrate noncompetitive and reversible inhibition of USP1/UAF1. In NSCLC cells, they increase the monoubiquitylation of PCNA and FANCD2 [40]. The combination of pimozide and the MAST1 inhibitor lestaurtinib markedly decreases MAST1 expression and the phosphorylation of MEK1 and ERK in cancer cells, enhancing their sensitivity to cisplatin [43]. In a model of rituximab/chemotherapy-resistant diffuse large B-cell lymphoma, pimozide synergizes with etoposide, destabilizing MAX, thereby inhibiting cell proliferation and inducing apoptosis, autophagy, and cell cycle arrest [257]. However, the interaction of pimozide and GW7647 with other proteins, independent of their DUB activity, may restrict their application in certain contexts.

The discovery of C527 and its more potent derivatives in 2013 marked a significant advancement, although their selectivity remains limited [263]. SJB3-019A, a derivative of C527, diminishes MM cell viability and mitigates resistance to BTZ. Its combinatory application with the HDAC inhibitor ACY-1215, BTZ, lenalidomide, or pomalidomide shows synergistic cytotoxic effects on MM cells [258]. Additionally, a new compound, ML323, surpasses GW7647 in terms of potency. With excellent selectivity against human DUBs, deSUMOylases, deneddylases, and unrelated proteases, ML323 boosts cytotoxicity in cisplatin-resistant NSCLC by blocking PCNA and FANCD2 deubiquitination [38, 264]. Moreover, ML323 selectively targets a subgroup of BRCA1-deficient cells that have developed resistance to PARP inhibitors through replication fork stabilization [143].

### USP13 inhibitors

USP13 inhibitors play a critical role in modulating DNA repair mechanisms. USP13 can deubiquitinate DNA topoisomerase 2 binding protein 1 (TopBP1), influencing DNA chain breakage and repair processes. Depleting USP13 enhances cellular sensitivity to replication stress inducers such as hydroxyurea (HU), camptothecin (CPT), ultraviolet (UV) radiation, and 5-Fu [265]. An imaging-based screening method led to the identification of spautin-1, a potent autophagy inhibitor that targets both USP10 and USP13 [266]. Spautin-1 disrupts RAP80-BRCA1 complex formation, impeding the DDR and enhancing the sensitivity of OC cells to olaparib. Combining spautin-1 with olaparib offers a superior synergistic therapeutic effect compared to olaparib alone [137]. In addition, spautin-1, when used with the EGFR inhibitor afatinib, significantly reduces the viability of NSCLC cells [197]. In a GIST cell-derived mouse xenograft model, spautin-1 triggers ATG5 degradation, and its use with 3-methyladenine notably enhances the therapeutic impact of IM [158].

### USP14 inhibitors

Compound b-AP15 is recognized for inducing apoptosis by targeting USP14 and UCHL5 [267]. It is particularly effective in inducing apoptosis in cells overexpressing BCL-2 or lacking functional p53, positioning it as a viable therapeutic approach for BTZ-resistant WM patients [259]. In 2015, the development of VX1570 improved the physicochemical properties of b-AP15 [268]. VX1570 prompts rapid, tumor-specific apoptosis in WM cells resistant to BTZ or ibrutinib, diminishing tumor load and extending survival in WM xenograft models [173]. A subsequent screening of 63,052 compounds identified a novel USP14 inhibitor, IU1, which specifically binds to the active form of USP14, inhibiting its association with the proteasome while sparing other DUBs [269]. When paired with anti-PD-1 therapy, IU1 markedly reduced tumor mass and extended survival in mouse models [228].

### USP8 inhibitors

DUBs-IN-2 is an effective USP8 inhibitor with potential in countering various types of immunotherapy resistance. Its application leads to PD-L1 upregulation, which stimulates immune responses and antigen presentation, thus transforming the TME into a more inflamed state. This alteration in TME bolsters the effectiveness of anti-PD-1/PD-L1 immunotherapy across several mouse tumor models [207]. In pancreatic cancer, the combination of DUBs-IN-2 and anti-PD-L1 therapy activates cytotoxic T cells, significantly inhibiting tumor growth [206]. Moreover, a synthesized USP8 inhibitor, 9-Ethyloxyimino-9H-indeno [1,2-b] pyrazine2,3-dicarbonitrile, has been shown to suppress multiple RTKs in gefitinib-resistant NSCLC cells. This inhibitor promotes the colocalization of ubiquitin and target RTKs, effectively overcoming gefitinib resistance in lung cancer [196]. Additionally, in HCC cells and mouse models, 9-Ethyloxyimino-9H-indeno [1,2-b] pyrazine2,3-dicarbonitrile significantly boosts the effectiveness of Dox or sorafenib by reducing RTK expression by approximately 90% [90].

## Conclusions and perspectives

In this review, we have thoroughly discussed the intricate mechanisms of USP-mediated drug resistance proceeding from the perspectives of various treatment strategies and specific drugs, and suggested that targeting USPs may offer novel insights into overcoming drug resistance in cancer therapy. Undoubtedly, USP inhibitors have the potential to counteract drug resistance and enhance the responsiveness of cancer cells to anti-cancer treatments, including chemotherapy, molecular targeted therapy, immunotherapy, and radiotherapy. Although the primary focus of our review is to provide insights and perspectives for clinical treatment by exploring USP-mediated cancer therapy resistance within the context of different clinical approaches, it is important to note the inherent interconnectedness between different USPs and drug resistance mechanisms. For instance, USP7 has been implicated in promoting DDR, thus mediating resistance to DNA-damaging chemotherapeutic agents and also radiation therapy. Furthermore, as one of the most extensively studied USPs, USP7 is not only involved in DDR but also participate in EMT, CSC generation, anti-apoptosis, hypoxia, angiogenesis, and modulation of immune cell infiltration within the TME. These biological functions collectively contribute to the development of resistance mechanisms in cancer therapy. Therefore, USP7 mediates resistance to a wide range of chemotherapeutic agents, radiation therapy, and immunotherapy. Another notable example is USP22, which significantly impacts the efficacy of chemotherapy drugs and immunotherapy due to its involvement in EMT, CSC formation, and modulation of TME. The overarching framework of this review focuses on the interaction between USPs and drugs, with a specific emphasis on USP vs. cellular pathway/functional signaling within each particular drug category. Different drug action mechanisms determine the specific resistance signaling mechanisms mediated by USPs, while the USP-mediated signaling pathways, in turn, contribute to varied drug resistance profiles. These relationships exhibit overlapping and reciprocal influences.

Therefore, expanding on these aspects not only deepens the understanding of the complex dynamics underlying USP-mediated resistance but also sheds light on the challenges faced by researchers aiming to unravel these intricate networks and optimize therapeutic outcomes. Given the complexity of USP regulatory network, the exact mechanisms by which USP inhibition can be leveraged to surmount resistance to anti-cancer drugs remain incompletely elucidated. While USPs have demonstrated potential in mediating cancer drug resistance, several challenges and considerations must be addressed.

**Fig. 6:**
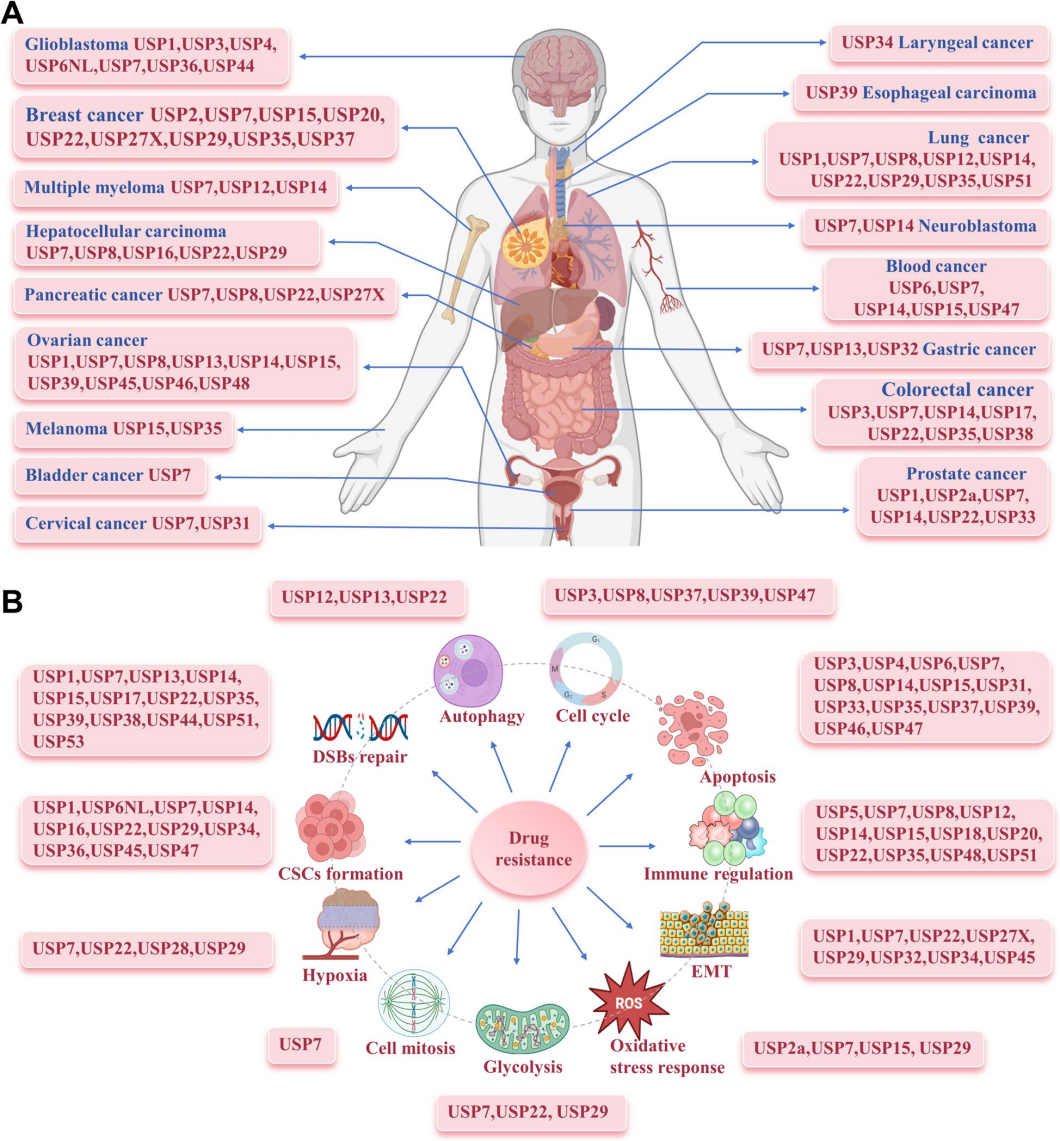
USPs exhibit overlapping expressions and functional mechanisms in mediating drug resistance during cancer treatment. **A** The expression and variability of USPs contribute to drug resistance across various cancer types. **B** Different USPs orchestrate drug resistance through intricate functional mechanisms

Firstly, we catalogued the USPs implicated in cancer drug resistance across various cancer types, as illustrated in Fig. 6A. The expression patterns of USPs across different cancer types reflect specific molecular alterations and signaling pathways of each cancer. The presence of multiple USPs within a particular cancer type or the expression and variation of same USP (e.g., USP7, USP14, and USP22) across different cancers suggest functional redundancy. This implies that different USPs may substitute for one another's functions and substrates, adding to their role complexity in cancer and complicating the targeting of a singular USP for treatment. Tumors consist of a heterogeneous mix of cancer cells, each with unique genetic and phenotypic characteristics. Within a tumor, cancer cells can have diverse molecular signatures, including USP expression variations. The impact of USPs on drug resistance is context-dependent, shaped by the specific cellular environment, TME, and genetic landscape, which can also shift in response to external stimuli, such as environmental changes or treatment. This variability introduces further complexity in pinpointing the precise USPs responsible for cancer drug resistance. Overcoming these challenges necessitates extensive profiling of USP expressions and activities across a range of cancer types and stages to track USP dynamics and comprehend their roles. Traditional methods for USP activity assessment, like biochemical assays, may not suit clinical samples or lack necessary sensitivity and specificity. Thus, developing precise and reliable assays for measuring USP activity in patient-derived samples is crucial for identifying USPs pivotal in drug resistance. This endeavor often requires merging multi-omics data, including genomics, transcriptomics, proteomics, and epigenomics to pinpoint USPs linked to drug resistance in particular cancer scenarios. Fostering interdisciplinary collaboration, employing advanced technologies, and analyzing extensive patient cohorts could lead to personalized treatment strategies targeting specific USPs involved in drug resistance for each cancer type.

**Fig. 7:**
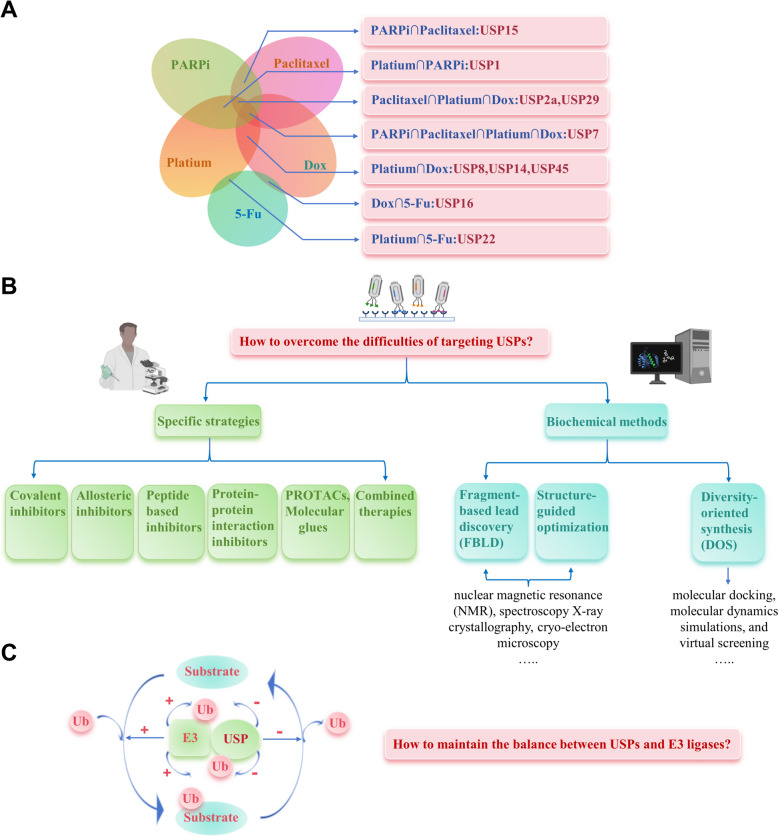
Targeting USPs is challenging within the complex UPS to overcome drug resistance. **A** The overlapping expressions and regulatory functions of USPs are illustrated in mediating drug resistance among different DNA-damage inducing agents. **B** The essential strategies and specific biochemical approaches are crucial in addressing the challenges of targeting USPs. **C** The interaction between USPs and E3 ligases maintains the equilibrium between ubiquitination and deubiquitination process in drug resistance

Secondly, the USP family consists of numerous members with overlapping function in drug resistance (Fig. 6B). Abovementioned USP7 and USP22 has been implicated in mediating drug resistance through a variety of molecular mechanisms, thus USP7 and USP22 can affect the efficacy of multiple drugs, not just one specific drug (Fig. 7A). In addition, USP2a and USP29 have been shown to influence the therapeutic resistance to paclitaxel, platinum, and Dox. The overlapping functions of different USPs in cancer drug resistance pose challenges for achieving functional specificity and avoiding cross-influence. While some USPs may confer “drug-resistance” roles by stabilizing crucial signaling proteins, others may function as suppressors through deubiquitinating and activating proteins in various molecular pathways. Although multiple USPs may participate in the same cellular processes or drug strategies, they often have unique substrates or regulatory networks that bestow specific resistant functions. Identifying the precise molecular mechanisms that underpin the drug resistance-associated functions of individual USPs is crucial, necessitating a blend of experimental and computational methods. Functional studies, such as RNA interference and CRISPR/Cas9-mediated gene knockout, can modulate the expression of specific USPs in cancer cells or animal models during drug treatment. High-throughput screening can identify downstream substrates or binding partners of USPs relevant to drug resistance. Biochemical assays, like in vitro deubiquitination assays using recombinant USPs and targets, can elucidate specific protein targets and deubiquitination events. These assays, combined with drug treatments, assess the impact of USPs on drug responses. Importantly, computational modeling, including molecular dynamics simulations, docking studies, or network analysis, can predict and elucidate interactions between USPs, their substrates, and drug resistance molecules. Network-based approaches can identify crucial nodes or modules within signaling networks affected by USPs in the context of drug resistance.

Thirdly, although the identification and creation of strong and specific USP inhibitors is an exciting field with promise for improving cancer outcomes, the challenge of targeting USPs persists. USPs possess conserved catalytic domains, with the active site of USPs featuring a catalytic triad in the Cys and His domains, comprising a cysteine residue, a histidine residue, and an aspartate residue. These residues are essential for the deubiquitinating activity of USPs. The task of designing inhibitors that can specifically target the active site and hinder the catalytic activity of USPs is daunting due to the high conservation of the catalytic triad across various USPs. Additionally, the catalytic domain of USPs can demonstrate structural flexibility, enabling the accommodation of a wide range of substrates. This adaptability presents obstacles in developing small molecule inhibitors that can selectively target individual USPs without impacting other USPs with similar structures. Consequently, many existing USP inhibitors may exhibit off-target effects, affecting cellular processes unrelated to drug resistance, and limiting the therapeutic potential of USP inhibitors. We posit that researchers are pursuing various strategies to address the challenges of targeting USPs (Fig. 7B). 1) Covalent inhibitors: The development of covalent inhibitors that form irreversible bonds with the USP active site can enhance both selectivity and potency. These inhibitors target unique reactive residues within the USP active site for more effective and specific inhibition. 2) Allosteric inhibitors: Instead of the catalytic site, allosteric inhibitors attach to different sites on the USP protein, altering its activity. This method aims to achieve selectivity by focusing on distinctive conformational states or regulatory regions of the USP. 3) Peptide-based or protein-protein interaction inhibitors: Inhibitors derived from USP substrates or interacting proteins can disrupt USP's interactions with its substrates or regulatory proteins, thereby inhibiting its activity. 4) PROTACs and molecular glues: These innovative strategies employ bifunctional molecules to direct USPs towards an E3 ubiquitin ligase or a target protein for ubiquitination and subsequent degradation. This leverages the proteasomal degradation pathway to indirectly diminish USP levels and their activity. 5) Combination therapies: To address potential resistance to USP inhibitors, similar to other targeted therapies, their combination with other treatments could enhance therapeutic efficacy and potentially forestall or delay resistance development. Furthermore, with advancements in science and technology, increasingly sophisticated drug design strategies are being applied (Fig. 7B). It's critical to acknowledge that these strategies are in active research phases, and their success may vary by the specific USP and disease context. Ongoing research in these fields is promising for surmounting USP targeting challenges and improving therapeutic outcomes. We maintain that through the integration of computational modeling and synthesis, USP inhibitors can be identified and optimized to achieve enhanced potency, selectivity, drug-like characteristics, and minimized off-target effects.

Lastly, despite the existence of over 1,000 E3 ligases, there are fewer than 100 DUBs, with USPs constituting a significant subgroup. This discrepancy in numbers suggests that USPs perform multiple roles and participate in various cellular processes critical for overcoming drug resistance. The interactions and competition between E3 ligases and USPs represent a pivotal area of research (Fig. 7C). E3 ligases facilitate ubiquitination and protein degradation, whereas USPs reverse this by deubiquitinating the same substrates, allowing for precise regulation of ubiquitylation status, which affects the stability, localization, and activity of substrate proteins in cancer therapy. Beyond targeting the same protein substrates for their antagonistic effects, USPs and E3 ligases can interact directly, acting as substrates for each other's ubiquitination or deubiquitination activities. Notably, many USPs are linked with E3 ligases, such as USP7 and MDM2, USP15 and Keap1, USP47 and β-Trcp, which are prone to self-ubiquitylate or even to enhance the ubiquitination activities of E3 ligases through deubiquitinating them. Hence, the stabilization of E3 ligases through deubiquitination underscores a key aspect of USP function, while E3 ligases may destabilize their corresponding USPs via ubiquitination. This raises the question of whether specific research strategies could modulate the interaction and competitive dynamics between USPs and E3 ligases to improve the targeting of USPs in drug resistance. Investigating potential reciprocal regulatory networks and biomarkers between USPs and E3 ligases related to drug resistance, are critical initial steps. These efforts can inform drug design and targeted therapy. By precisely mapping interaction sites and employing computational methods, novel drugs could be developed to either simultaneously sensitize E3 ligase and inhibit USP or co-inhibit both, enabling dual or multiple substrate protein degradation for augmented anti-cancer effects. However, strategies to regulate USP and E3 ligase interactions are still nascent, necessitating further research to confirm their efficacy and safety. Sensitizing or inhibiting specific E3 ligases might also trigger unintended degradation of unknown substrate proteins, highlighting the need for additional investigation and consideration.

It is crucial to emphasize that, while detailed mechanistic roles have shown promise in preclinical studies, the clinical validation of USP inhibitors for overcoming drug resistance remains nascent, necessitating further investigation into their safety, efficacy, and long-term impacts. Executing well-designed clinical trials with meticulous patient selection and rigorous outcome measures is vital to ascertain the clinical utility of USP inhibitors in combating drug resistance. The identification and validation of predictive biomarkers that can categorize patients based on their likelihood of responding to USP inhibitors will enhance patient selection and facilitate monitoring of treatment responses.

In conclusion, this review advances current understanding of USPs' complex roles, suggesting that targeting USPs could be a strategic approach to tackling tumor resistance. It may also uncover new clinical applications and provide a framework for the future improvement of USP inhibitors. However, comprehensive research is required to elucidate the complex mechanisms by which USPs influence drug resistance. This includes additional studies to decipher the USP family's complexity and redundancy, develop personalized treatment modalities based on tissue-specific USP profiling, enhance the selectivity and specificity of USP inhibitors, investigate combination therapies to circumvent resistance, and implement rigorous clinical trials with strategic patient selection and biomarker validation. By confronting these challenges, the potential of USPs as therapeutic targets for countering drug resistance in cancer can be more fully realized.

## Data Availability

No datasets were generated or analysed during the current study.

## Abbreviations

PTM: Post-translational modification
DUB: Deubiquitinating enzyme
UPS: Ubiquitin proteasome system
USP: Ubiquitin-specific protease
UBL: Ubiquitin-like
ZnF-UBP: Zinc finger ubiquitin-binding
DUSP: Domains specific to USP
UIM: Ubiquitin-interacting motifs
UBA: Ubiquitin-associated
CSC: Cancer stem cell
EMT: Epithelial mesenchymal transformation
DDR: DNA damage response
CHK1: Checkpoint kinase 1
DSBs: Double strand breaks
PALB2: partner and localizer of BRCA2
CtIP: C-terminal binding protein-interacting protein
NSCLC: Non-small cell lung cancer
UAF1: USP1 associated factor 1
ZEB1: Zinc finger E-box binding homeobox 1
OC: Ovarzian cancer
ESCC: Esophageal squamous cell carcinoma
PARP: Poly‑ADP ribose polymerase
IAP: Inhibitor of apoptosis protein
SIRT1: Sirtuin 1
TNBC: Triple-negative breast cancer
ALDH: Aldehyde dehydrogenase
SOC: Serous ovarian cancer
BC: Breast cancer
CAF: Cancer-associated fibroblast
GC: Gastric cancer
LSCC: Laryngeal squamous cell carcinoma
5-Fu: 5-fluorouracil
lncRNA: Long non-coding RNA
CRC: Colorectal cancer
HIF-1α: Hypoxia-inducible factor
AR: Androgen receptor
HCC: Hepatocellular carcinoma
FUCA1: α-L-fucosidase 1
NER: Nucleotide excision repair
HDAC3: Histone deacetylase 3
Dox: Doxorubicin
TME: Tumor microenvironment
PDAC: Pancreatic ductal adenocarcinoma
NB: Neuroblastoma
β-Trcp: β-transducin repeat-containing protein
RTK: Receptor tyrosine kinase
ABC: ATP-binding cassette,
Pgp: P-glycoprotein
Ct-HBx: Carboxyl-terminal truncated form of the Hepatitis B virus X protein
CAM-DR: Cell adhesion-mediated drug resistance
MM: Multiple myeloma
ROS: Reactive oxygen species
CRPC: Castration-resistant prostate cancer
JNK: cJUN NH2-terminal kinase
OS: Oxidative stress
Nrf2: NF-E2-related factor 2
lipid-ROS: Lipid reactive oxygen species
hnRNPA1: Heterogeneous nuclear ribonucleoprotein A1
ALOX15: Arachidonate lipoxygenase 15
TS: Thymidylate synthase
PcG: Polycomb group
HNSCC: Head and neck squamous cell carcinoma
MRP1: Multidrug resistance-associated protein 1
GBM: Glioblastoma
GSC: Glioma stem cell
USP6NL: USP 6 N-terminal-like protein
CAV1: Caveolin-1
SSBs: Single strand breaks
NHEJ: Nonhomologous end-joining
HR: Homologous recombination
RAP80: Receptor-associated protein 80
TLS: Translesion synthesis
FBP1: Fructose-1,6-bisphosphatase 1
DNMT1: DNA (cytosine-5)-methyltransferase 1
TKI: Tyrosine kinase inhibitor
IM: Imatinib
CML: Chronic myeloid leukemia
GIST: Gastrointestinal stromal tumor
PBMC: Peripheral blood mononuclear cell
GLS1: Glutaminase-1
hucMSC: Human umbilical cord mesenchymal stem cell
YB-1: Y-box binding protein 1
ATG5: Autophagy-related protein 5
METTL3: N6-methyladenosine methyltransferase-like 3
Rab35: Ras-related protein Rab-35
Gal-SLP: Galactose-decorated lipopolyplex
PDX: Patient-derived xenograft
HK2: Hexokinase 2
BTK: Bruton's tyrosine kinase
BCR: B-cell receptor
CLL: Chronic lymphocytic leukemia
WM: Waldenstrom macroglobulinemia
ADT: Androgen deprivation therapy
HSPC: Hormone-sensitive prostate cancer
KIF15: Kinesin family member 15
GRP75: Glucose-regulated protein 75
HSP: Heat shock protein
SIX1: Sine oculis homeobox homolog 1
ER: Estrogen receptor
OS: Overall survival
T-DM1: Ado-trastuzumab emtansine
PI: Proteasome inhibitor
BTZ: Bortezomib
HMGB1: high mobility group box-1
PD-1: Programmed death protein-1
PD-L1: Ligand programmed death-ligand
CTLA-4: Cytotoxic T lymphocyte-associated antigen-4
GSDME: Gasdermin E
TAMs: Tumor-associated macrophages
ISGs: IFN-stimulated genes
TET2: Ten-eleven translocation 2
IMiDs: Immunomodulatory drugs
PROTACs: Proteolysis-targeting chimeras
TRP: tryptophan
KYN: Kynurenine
IDO1: Indoleamine 2,3-dioxygenase 1
IR: Ionizing radiation
DDB2: DNA damage binding protein 2
TopBP1: Topoisomerase 2 binding protein 1
HU: Hydroxyurea
CPT: Camptothecin
UV: Ultraviolet
